# The Peptide PROTAC Modality: A New Strategy for Drug Discovery

**DOI:** 10.1002/mco2.70133

**Published:** 2025-03-24

**Authors:** Youmin Zhu, Yu Dai, Yuncai Tian

**Affiliations:** ^1^ Shanghai AZ Science and Technology Co., Ltd. Shanghai China; ^2^ School of Biotechnology East China University of Science and Technology Shanghai China

**Keywords:** peptide PROTAC, drug discovery, precision medicine

## Abstract

In recent years, proteolysis targeting chimera (PROTAC) technology has made significant progress in the field of drug development. Traditional drugs mainly focus on inhibiting or activating specific proteins, while PROTAC technology provides new ideas for treating various diseases by inducing the degradation of target proteins. Especially for peptide PROTACs, due to their unique structural and functional characteristics, they have become a hot research topic. This review provides a detailed description of the key components, mechanisms, and design principles of peptide PROTACs, elaborates on their applications in skin‐related diseases, oncology, and other potential therapeutic fields, analyzes their advantages and challenges, and looks forward to their future development prospects. The development of peptide PROTAC technology not only opens up new paths for drug research and development, but also provides new ideas for solving the resistance and safety issues faced by traditional small‐molecule drugs. Compared with small‐molecule PROTACs, peptide PROTACs have advantages such as multitargeting, biodegradability, low toxicity, and flexibility in structural design. With the deepening of research and the continuous maturity of technology, peptide PROTACs are expected to become one of the important strategies for future drug discovery, providing new hope for the treatment of more intractable diseases. Peptide PROTACs are ushering in a new era of precision medicine.

## Graphical abstract

Peptide PROTAC is a bifunctional molecule generally composed of a target protein binding peptide (TBP), a linker peptide (LP), an E3 ligase binding peptide (EBP), and a delivery‐enhancing peptide (DEP), which can bind to the target protein (P) and E3 ligase (E3), inducing P to be labeled with ubiquitin (Ub) and subsequently recognized and degraded by the proteasome (PS)

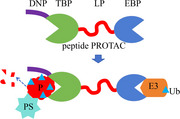

## 1 Introduction

In the past few years, proteolysis targeting chimera (PROTAC) technology has become a popular research direction in the field of biotechnology. In 2001, Sakamoto et al. [1] first proposed the concept of PROTAC, which laid the foundation for the subsequent development of PROTAC technology. In 2013, Arvinas was founded, becoming the first biotechnology company to focus on PROTAC technology, which greatly accelerated the commercialization of PROTAC technology [2]. In 2022, the first PROTAC drug ARV‐110 achieved initial success in clinical trials, further demonstrating the feasibility and effectiveness of PROTAC technology [3].

Compared with traditional drug treatment methods, PROTACs can more effectively overcome resistance issues because they can degrade the entire pathogenic protein without compensatory increases or mutations [4]. For example, after receiving treatment with the Bruton tyrosine kinase (BTK) inhibitor ibrutinib, over 80% of patients with chronic lymphocytic leukemia developed C481S mutations, leading to drug resistance. Fortunately, a series of PROTACs (MT‐802, SJF620, and L18I) can effectively degrade various BTK mutations and overcome ibrutinib resistance induced by BTK mutations [5]. In theory, PROTAC‐targeted protein degradation technology can be applied to any field involving intracellular proteins [6]. However, the full potential of this technology in the field of disease treatment has yet to be realized. If PROTAC technology is applied to the development of drugs for disease treatment, it can degrade certain specific proteins in the body, thereby achieving targeted treatment of tumors, skin diseases, and other stubborn diseases. This highly specific and efficient protein degradation method offers a new approach to addressing deeply rooted problems that are difficult to treat with traditional methods. Meanwhile, traditional techniques may find it difficult to identify drug targets, but PROTAC technology can create more targets, turning previously “untreatable” areas into usable targets [7]. PROTAC molecules have high specificity, ensuring that they can accurately recognize the target proteins without affecting nontarget proteins. PROTAC molecules exert their effects by degrading target proteins, enabling their effectiveness to be superior to that of inhibition [8]. Once the target protein is successfully degraded, PROTAC molecules are released from the complex. The released PROTAC molecules can continue to bind to the next target protein and enter the next degradation cycle, making them reusable. Thus, very low doses of PROTAC molecules can achieve strong degradation effects [9]. Additionally, PROTAC technology can accurately target and remove aging and damaged proteins without affecting normal proteins and physiological functions [10].

Currently, most PROTACs are small chemical molecules. However, a small‐molecule PROTAC generally acts on only one target, with poor biodegradability, high toxicity, and inflexibility of structure design [11]. With the rapid development of structural biology, it is becoming increasingly convenient to obtain peptides with high affinity for targets, which provides possibilities for the design and development of peptide PROTACs. The development of peptide PROTACs typically requires detailed information on the structure, function, and interaction interfaces of the proteins involved in the interaction. With the advancement of computing technology and the development of bioinformatics, the design and optimization of peptide PROTACs have become more efficient, making their targets more numerous and precise than those of small‐molecule PROTACs [12]. Unlike small‐molecule PROTACs, peptide PROTACs can act on multiple targets by concatenating multiple different peptide segments. Peptide PROTAC utilizes the body's own biological mechanisms to act, reducing the risk of side effects of traditional skin disease treatment drugs [13]. Peptide PROTAC exhibits good biocompatibility when in contact with the human body, reducing the risk of immunogenicity and toxic reactions, ensuring safety during use. Due to the safety of peptide PROTAC, it can be safely applied to the human body. For example, Zhang et al. [14] have developed a peptide PROTAC drug targeting p300, which can effectively degrade p300 and kill cancer cells in castration‐resistant prostate cancer (CRPC), androgen receptor (AR)‐negative, and neuroendocrine prostate cancer (NEPC) cells, greatly promoting the development of precision medicine. This biological safety guarantee makes the application of peptide PROTAC in precisely targeted disease treatment more reliable and reassuring. Patients can obtain the efficient disease treatment effects it brings without worrying about potential safety hazards. With the advancement of science and technology, people's requirements for disease treatment drugs are no longer limited to basic symptom improvement, but pay more attention to their precision, safety, and effectiveness. Peptide PROTAC technology, as an emerging biotechnology, has shown great potential in the field of disease treatment and deserves further exploration [15].

This review elaborates on the composition, design methods, and mechanisms of peptide PROTACs, with a focus on their potential applications in skin‐related diseases, oncology, and other potential therapeutic fields. It discusses the challenges faced by peptide PROTACs in synthesis and purification, stability and bioavailability, production cost and pricing, regulation and approval, and looks forward to the future prospects of peptide PROTAC research and development, including innovation in peptide design and delivery systems, clinical translation, and therapeutic potential, as well as emerging trends and market prospects, providing useful reference for researchers and enterprises in the disease treatment field. With the continuous deepening of research on peptide PROTAC technology and its expanding application in the field of disease treatment, we believe that this innovative technology will bring safer, more efficient, and personalized solutions for precisely targeted disease treatment. In the future, peptide PROTAC technology may lead the disease treatment field into a new stage of development, enabling patients to receive precise and effective targeted therapy.

## 2 Overview of PROTAC and Peptide PROTAC Molecules

### 2.1 PROTAC Hybrid Bifunctional Molecules

**FIGURE 1:**
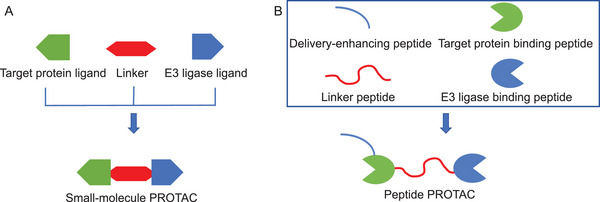
Schematic structure of small‐molecule PROTAC and peptide PROTAC. (A) Small‐molecule PROTAC is composed of a target protein ligand, a linker, and an E3 ligase ligand. (B) Peptide PROTAC is composed of a delivery‐enhancing peptide, a target protein binding peptide, a linker peptide, and an E3 ligase binding peptide.

PROTAC hybrid bifunctional molecules are a novel target protein degradation design strategy based on a protein degradation system. They consist of three main components: a ligand that can specifically bind to the target protein, a ligand that can bind to E3 ubiquitin (Ub) ligase, and a linker that connects the two [16]. These hybrid molecules form a ternary complex by simultaneously binding the target protein and E3 Ub ligase, guiding the target protein into the ubiquitination‐proteasome system (UPS) and achieving its specific degradation (Figure 1).

The target protein ligand is the part of a PROTAC molecule responsible for recognizing and binding to the target protein. It is usually a small‐molecule compound with high specificity and affinity for target proteins, capable of accurately identifying and binding to specific target proteins within cells. The design and screening of target protein ligands are typically based on known bioactive molecules or through in‐depth analysis of the target protein structure [17]. Researchers can obtain target protein ligands with the desired activity through chemical synthesis or biological screening. The linker is a bridge in PROTAC molecules that connects the target protein ligand with the E3 ligase ligand. It must be flexible enough to ensure that the target protein ligand and E3 ligase ligand can have a certain degree of mobility; at the same time, it must also be stable enough to maintain the overall structure of the PROTAC molecule. There are various types of connectors, such as straight‐chain alkanes. Chain length is an important parameter in linker design. A chain that is too short may hinder the formation of ternary complexes due to spatial collisions, while a chain that is too long may increase binding entropy and reduce binding efficiency [18]. The E3 ligase ligand is the part of the PROTAC molecule responsible for recruiting intracellular E3 Ub ligases. E3 Ub ligases are key enzymes in the UPS, responsible for labeling target proteins with Ub molecules and triggering their degradation [19]. There are various types of E3 ligase ligands, and different E3 ligase ligands can recruit different types of E3 Ub ligases. Common E3 ligase ligands include cereblon (CRBN) ligands based on pomalidomide and VHL ligands based on thalidomide [20].

Professor Hou Tingjun from Zhejiang University has established the PROTAC information database PROTAC‐DB (http://cadd.zju.edu.cn/protacdb/compound). This database includes a great deal of structural information and experimental data on PROTACs [21]. By November 2024, they had already collected at least 6111 PROTACs, 569 warheads (small molecules targeting proteins of interest), 107 E3 Ub ligase ligands, 2753 linkers, and 959 ternary models, as well as the chemical structures, biological activities, physicochemical properties, degradation abilities, binding affinities, and cellular activities of the PROTAC molecules.

### 2.2 Comparison Between Small‐Molecule and Peptide PROTACs

Small‐molecule PROTAC and peptide PROTAC are the two main types in PROTAC technology, each with its own characteristics in structure and function. Small‐molecule PROTAC is typically composed of two small‐molecule ligands connected by a chemical linker, which can be known drug molecules or synthesized compounds. The molecular weight of small‐molecule PROTACs is relatively small, and their composition generally includes a target protein ligand, a linker, and an E3 ligase ligand. Small‐molecule PROTACs generally require multiple chemical synthesis steps, which can be relatively cumbersome. The linker of small‐molecule PROTACs has a significant impact on their permeability. Small‐molecule PROTACs need to maintain good solubility in aqueous environments in order to be effectively distributed and metabolized in the body. The substitution of hydrophilic functional groups may improve the water solubility of small‐molecule PROTACs, thereby enhancing their bioavailability. However, the substitution of hydrophilic functional groups may reduce the affinity of small‐molecule PROTACs. The target protein ligand of small‐molecule PROTACs determines their targeting ability. The target protein ligands and E3 Ub ligase ligands jointly determine their affinity [4] (Table 1).

**TABLE 1:** Comparison between small‐molecule and peptide PROTACs.

	Small‐molecule PROTACs	Peptide PROTACs
Essence	Chemical compound [4]	Peptide [4]
Molecular weight	Small [4]	Medium [4]
Synthesis method	Chemical synthesis [4]	Biological synthesis [4]
Synthesis steps	Multiple, tedious [4]	Once, simple [4]
Composition	Target protein ligand, linker, E3 ligase ligand [4]	Target protein binding peptide, linker peptide, E3 ligase binding peptide, and delivery‐enhancing peptide [4]
Permeability	Related to the length of the linkers [4]	Related to delivery‐enhancing peptide [4]
Implementation of hydrophilicity	Substitution of hydrophilic functional groups [4]	Substitution of hydrophilic amino acids [4]
Targeted determination	Target protein ligand [4]	Target protein binding peptide [4]
Affinity determination	Target protein ligand, E3 ligase ligand [4]	Target protein binding peptide, E3 ligase binding peptide [4]

The peptide segments of PROTACs typically originate from known protein–protein interaction (PPI) interfaces. Peptide PROTACs have a relatively medium molecular weight and are generally composed of a target protein binding peptide, a linker peptide, and an E3 ligase binding peptide. Peptide PROTACs can be synthesized directly through gene expression, with simple steps. To improve the cell membrane or skin permeability of peptide PROTACs, a delivery‐enhancing peptide is usually added. The water solubility of peptide PROTACs can be achieved through hydrophilic amino acid substitution. In theory, the function of peptide PROTACs remains unchanged after the substitution of structurally similar amino acids, but their hydrophilicity will change. The target protein binding peptide and E3 ligase binding peptide of peptide PROTACs are related to their affinity, and the target protein binding peptide determines their targeting ability [4] (Table 1).

### 2.3 Advantages of Peptide PROTACs

The targeting ability of peptide PROTACs is mainly due to their unique structural design and highly specific peptide chains. The design of peptide chains is typically based on known PPI sites, which have highly conserved structural features on the target protein. By accurately identifying these sites, peptide PROTACs can efficiently bind to target proteins, thereby achieving highly specific targeting effects. The high targeting ability of peptide PROTACs is also reflected in their ability to degrade proteins that traditional drugs find difficult to target. Many disease‐related proteins are considered to be “untargetable” due to conformational reasons or lack of binding sites. However, peptide PROTACs provide new possibilities for treating these diseases by inducing ubiquitination and degradation of these proteins (Table 2) [22].

**TABLE 2:** Advantages of peptide PROTACs.

	Advantages of peptide PROTACs
Targeting	High targeting and specificity; targeting proteins that were originally considered “untargetable”' [22]
Affinity	High affinity; effectively binding to target proteins at low concentrations [22]
Biological activity	High biological activity; a small amount of peptide PROTAC molecules can trigger the degradation of a large amount of target proteins [22]
Multitarget degradation	Connecting multiple peptide segments to achieve the degradation of multiple target proteins [22]
Flexibility in structural design	High flexibility in structural design; the length and sequence of peptide segments can be precisely designed, and the peptide segments can be flexibly selected [22]
In vivo half‐life	Long; with high polarity and medium molecular weight [22]
Bioavailability	High; enriched inside the cell; high intracellular concentration; effectively utilized [22]
Toxicity	Low; reducing damage to normal tissues; can be degraded after completing their mission [22]
Plasticity	Easy to be modified; can be tracked and located [22]

Peptide PROTACs have high affinity. High‐affinity peptide chains can more effectively recruit E3 Ub ligases, promoting the ubiquitination and degradation of target proteins. In cells, target proteins often interact with other biomolecules, such as proteins and nucleic acids, to form complex networks. Peptide PROTACs, with their high affinity, can accurately locate and bind to target proteins in this complex biological environment, thereby achieving effective degradation of target proteins. The structural design of peptide chains enables them to quickly bind to target proteins upon entering cells, improving the efficiency of peptide PROTACs and reducing waiting times. High affinity not only ensures that peptide PROTACs can effectively bind to target proteins at low concentrations, but also reduces the dosage of drugs used and minimizes potential side effects (Table 2) [22].

Peptide PROTACs have relatively high biological activity. Through molecular dynamics simulations and structural biology techniques, researchers can accurately predict and optimize the conformation of peptide chains, enabling them to bind more effectively to target proteins and enhance their biological activity. Peptide PROTACs do not need to continuously occupy the active site of the target protein like traditional small‐molecule inhibitors. A small amount of peptide PROTAC molecules can trigger the degradation of a large number of target proteins, displaying strong activity. Peptide PROTACs can profoundly alter intracellular signaling pathways, metabolic processes, as well as cell proliferation, differentiation, and apoptosis functions by degrading target proteins, thereby exerting a strong intervention effect on the biological behavior of the entire cell [4].

Peptide PROTACs can achieve multitarget simultaneous degradation. Through clever molecular design, peptide segments targeting multiple targets can be linked together in the same peptide PROTAC molecule, enabling the simultaneous degradation of multiple target proteins closely related to diseases. This ability is particularly important in the treatment of complex diseases, as the occurrence and development of many diseases often involve the abnormal expression and interaction of multiple proteins. By simultaneously degrading these target proteins, peptide PROTACs have the potential to more effectively intervene in disease progression and provide patients with a more comprehensive treatment plan [4].

Peptide PROTACs have greater flexibility in structural design. The length and sequence of peptide segments can be finely adjusted to optimize their binding ability with target proteins. This flexibility allows researchers to design peptide PROTACs with optimal performance based on different target proteins and disease requirements. Different target protein binding peptides can be designed for different parts of the same target protein. Researchers can flexibly select suitable target protein binding peptides based on the structure and characteristics of the target protein to enhance the binding affinity and selectivity of peptide PROTACs. The linker peptide is a key component in peptide PROTACs that connects two ligands. Its length, flexibility, and chemical properties can be flexibly designed and adjusted. The E3 ligase binding peptides in peptide PROTACs can selectively bind to different E3 Ub ligases. Researchers can select the most suitable E3 ligase binding peptide based on the degradation pathway of the target protein and the intracellular UPS to improve the degradation efficiency and specificity of peptide PROTACs (Table 2) [23].

Peptide PROTACs exhibit significant advantages in prolonging the half‐life in vivo. Traditional small‐molecule drugs often have a short half‐life due to rapid metabolism and clearance, requiring frequent administration to maintain therapeutic efficacy. Peptide PROTACs typically have higher polarity and medium molecular weight, allowing them to effectively prolong their residence time in the body (Table 2) [4].

Peptide PROTACs also perform well in improving bioavailability. Bioaccumulation refers to the proportion of a drug that can be effectively utilized after entering the bloodstream, and is an important indicator for evaluating drug efficacy. The introduction of a delivery‐enhancing peptide increases its ability to penetrate the cell membrane, making it easier to be enriched inside the cell. Peptide chains can also promote drug endocytosis by interacting with cell surface receptors, further increasing their intracellular concentration. This mechanism not only improves the bioavailability of the drug, but also enhances its distribution in target tissues (Table 2) [4].

Peptide PROTACs exhibit significant advantages in reducing systemic toxicity. Traditional chemical drugs and small‐molecule inhibitors often have high systemic toxicity, mainly because they are widely distributed in the body, acting not only on target cells, but also potentially causing damage to normal cells. Peptide PROTACs, through their unique dual‐functional structure, can more accurately target disease‐related proteins, thereby reducing toxic side effects on normal cells. In addition, peptide PROTACs are essentially peptides that are ultimately degraded into amino acids after completing their mission, and thus possess significant biological safety. For example, Hines et al. [23] found that intraperitoneal injection of a peptide PROTAC at a dose of 10 mg/kg/day (maximum tolerated dose) exhibited lower toxicity compared with a PI3K small‐molecule inhibitor (LY294002) (Table 2).

The amino acid sequence of peptide PROTAC molecules exhibits extremely high plasticity and can be modified through simple chemical reactions to introduce various functional groups, such as fluorescent labeling, biotin labeling, or drug‐loading groups. These modifications can not only enhance the biological activity of peptide PROTACs, but also be used to study their distribution and metabolic processes in cells, greatly improving scientists’ ability to track and locate peptide PROTAC molecules at the cellular level, and providing strong support for further exploration of their functional mechanisms in organisms (Table 2) [22].

## 3 Design and Structure of Peptide PROTACs

**FIGURE 2:**
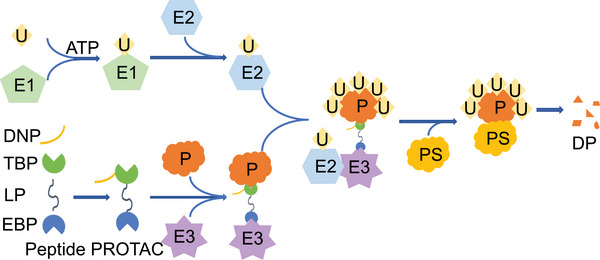
Diagram of the molecular mechanisms by which peptide PROTAC interacts with target proteins and E3 ligases. Ub utilizes ATP to bind to E1. E1 transfers the activated Ub to E2, and then E2 transfers Ub to E3. The bifunctional peptide PROTAC molecule binds to the target protein at one end and to E3 at the other end to form a ternary complex. E3 modifies the target protein through polyubiquitination. Subsequently, the polyubiquitination‐modified target protein is degraded by the proteasome. U, ubiquitin; E1, ubiquitin activating enzyme; E2, ubiquitin conjugating enzyme; E3, ubiquitin ligase; DNP, delivery‐enhancing peptide; TBP, target protein binding peptide; LP, linker peptide; EBP, E3 ligase binding peptide; P, target protein; PS, proteasome; DP, degraded protein.

In theory, peptide PROTAC should include at least three parts: target protein binding peptide, linker peptide, and E3 ligase binding peptide [24]. However, in order for the peptide PROTAC to be used as a disease treatment ingredient to enter cells and exert its effects, a delivery‐enhancing peptide is also needed (Figure 1) [25]. These four peptide segments work together to achieve functions such as penetrating into cells, binding to target proteins and E3 Ub ligases, and maintaining correct spatial conformation (Figure 2). The delivery‐enhancing peptide, target protein binding peptide, linker peptide, and E3 ligase binding peptide each have multiple options. By selecting different peptide segments and assembling them, multiple peptide PROTACs can be obtained.

### 3.1 Key Components of Peptide PROTACs

#### 3.1.1 Target Protein Binding Peptide

Target protein binding peptides are a key component of peptide PROTACs, responsible for recognizing and binding to specific target proteins. This binding process depends on the interaction between the target protein binding peptide and the target protein, which is usually based on hydrogen bonding, ionic bonding, or hydrophobic interactions between the peptide segment and the target protein. To ensure the specificity of peptide PROTACs, the design of target protein binding peptides requires precise matching of specific binding sites of the target protein to avoid nonspecific binding and potential side effects [26].

The binding ability of target protein binding peptides to target proteins affects the degradation efficiency of target proteins. By adjusting the amino acid sequence of the target protein binding peptide, its binding affinity for the target protein can be increased, thereby enhancing the degradation efficiency of the peptide PROTAC toward the target protein. In addition, optimizing the sequence of target protein binding peptides can better synergize with delivery‐enhancing peptides and E3 ligase binding peptides, thus improving the stability of the entire molecule.

#### 3.1.2 E3 Ligase Binding Peptide

The E3 ligase binding peptide fragment is responsible for binding to E3 Ub ligase, which can spatially bring E3 Ub ligase closer to the target protein, triggering the ubiquitination process of the target protein. The polyubiquitinated protein will be recognized and degraded by intracellular proteasomes. This process is a key step for peptide PROTACs to achieve specific target protein degradation [4]. This process involves three steps: Ub activation, Ub transfer, and Ub conjugation. Specifically, E3 Ub ligase can recognize and connect to a specific lysine of the target protein, adding Ub molecules to these lysine residues. When the substrate protein is ubiquitinated, it is sent to the proteasome complex for degradation. The E3 ligase binding peptide is involved in the recognition of substrate proteins, the regulation of Ub conjugation, and enzymatic hydrolysis. Several common E3 ligase binding peptides have good binding ability with E3 Ub ligases (Table 3) [4]. For example, Sakamoto et al. [1] discovered that a peptide (DRHDSGLDSM) could successfully induce ubiquitination. The minimum recognition sequence of HIF‐1α (ALAPYIP) has also been selected as an E3 ligase binding peptide, which can induce the degradation of HIF‐1α in vivo [27].

**TABLE 3:** Common E3 ligase binding peptides and linker peptides.

Category	Subclass	Sequences
E3 ligase binding peptide	–	RRRG [4]
		DRHDSGLDSM [1]
		ALAPYIP [27]
Linker peptide	Flexible connecting peptides	(G_4_S)*_n_* (*n* = 1–6) [30]
		(GSGS)*_n_* (*n* = 1–6) [30]
	Rigid connecting peptides	PAPAP [30]
		(EAAAK)*_n_* (*n* = 1–6) [30]
	Other linker peptides	SNAADEVATPEDVEPG [30]
Delivery‐enhancing peptides	TAT	YGRKKRRQRRR [37]
	Pep‑1	KETWWETWWTEWSQPKKKRKV [38]
	ANTP	RQIKIWFQNRRMKWKK [39]
	R9	RRRRRRRRR [40]
	TP10	AGYLLGKINLKALAALAKKIL [41]
	KALA	WEAKLAKALAKALAKHLAKALAKALKACEA [42]
	NLS	KRPAATKKAGQAKKKL [43]
	TD‐1	ACSSSPSKHCG [47]
	SPACE	ACTGSTQHQCG [48]
	Magainin	GIGKFLHSAKFGKAFVGEIMNS [49]

Abbreviations: KALA, klebsiella aerogenes alpha‐helical; NLS, nulear localization sequence; Pep‐1, permeating peptide 1; R9, arginine 9; SPACE, skin penetrating and cell entering; TAT, trans‐activator of transcription; TD‐1, transdermal peptide 1; TP10, transportan peptide.

The optimization of E3 ligase binding peptide sequence is a complex and meticulous process aimed at improving the binding efficiency between peptides and E3 Ub ligase, thereby regulating the ubiquitination process of proteins. First, understanding the interaction mechanism between E3 ligase binding peptides and E3 Ub ligase is crucial. This includes identifying key binding sites, interactions, and structural features [28]. Second, based on the known structure and substrate specificity of E3 Ub ligase, we can use computer simulation and bioinformatics tools to predict and optimize peptide sequences. These methods can help us screen peptide sequences with potential high binding affinity, reduce experimental workload, and improve success rates. For example, Ma et al. [29] used ProteinMPNN and RF diffusion to identify the binding peptides of the AR and Von Hippel Lindau (VHL), predicted their spatial relationship, and confirmed the binding ability of the designed peptide to AR and VHL. This provided a universal approach for the development of peptide PROTACs and potential therapeutic drugs for preventing androgenic alopecia [29]. In addition, considering the complexity and diversity of ubiquitination processes, we also need to increase the stability and activity of peptide sequences within cells by evaluating the expression level, half‐life, and interaction with other biomolecules of peptide sequences.

#### 3.1.3 Linker Peptides

Linker peptides are short peptides that act as linkers. The presence of linker peptides prevents the two peptides on both sides from folding or becoming entangled with each other, providing sufficient space for the two peptides to maintain their original stereoconformation and reducing the impact of steric hindrance on the structure and active centers of target protein binding peptides and E3 ligase binding peptides. The hydrophilicity and hydrophobicity of linker peptides are crucial for the stability of the functional domains of target protein binding peptides and E3 ligase binding peptides. Research has shown that the preferred amino acid residues for linker peptides are glycine, serine, proline, alanine, threonine, arginine, glutamine, and asparagine. Linker peptides are further divided into flexible connecting peptides and rigid connecting peptides. Flexible linking peptides are a class of amino acid sequences that are flexible, linear, and prone to bending. Some sequences rich in glycine or serine are more suitable as flexible linking peptides, such as (G_4_S)*_n_* (*n* = 1–6). Rigid linking peptides are a class of fixed‐length amino acid sequences that form a stable helical structure. PAPAP or (EAAAK)*_n_* (*n* = 1–6) is suitable as a rigid linker peptide (Table 3) [30].

The optimization of linker peptides is of great significance for improving the stability and activity of peptide PROTACs. By adjusting the length and flexibility of the linker peptides, they can better adapt to the spatial conformation between the target protein binding peptide and the E3 ligase binding peptide, thereby enhancing the stability of the entire molecule.

### 3.2 Role of Delivery‐Enhancing Peptides

The cell membrane, as the boundary of cells, forms a natural barrier with its special structure and biological function, which hinders therapeutic proteins, peptides, and oligonucleotides from reaching the site of action and exerting their pharmacological effects. To overcome this obstacle, delivery‐enhancing peptides have emerged. These peptides can help bioactive substances efficiently enter cells from outside, ensuring that they can reach their target sites and exert therapeutic effects. The delivery‐enhancing peptides provide a new pathway for drug delivery, improving the utilization and therapeutic efficacy of bioactive substances.

#### 3.2.1 Cell‐Penetrating Peptide

Cell‐penetrating peptides (CPPs) are a type of short peptides that can penetrate the cell membrane and enter the interior of the cell. These peptides typically consist of 5–30 amino acid residues and can efficiently enter cells without damaging the cell membrane structure. When CPPs are combined with drugs, their cell penetration ability can significantly improve the efficiency of drug delivery from the outside to the inside of the cell, thereby enhancing drug efficacy. CPPs have unique structures and chemical properties, which enable nonspecific interactions with the cell membrane and effectively penetrate it. This function is based on the lipophilicity and positive charge properties of CPPs, which enable CPPs to interact with the phospholipid bilayer on the cell membrane [31]. At present, the CPPs that have been extensively studied mainly include trans‐activator of transcription (TAT), permeating peptide 1 (Pep‐1), penetratin (ANTP), arginine 9 (R9), transportan peptide (TP10), klebsiella aerogenes alpha‐helical (KALA), and nuclear localization sequence (NLS), which can be used to deliver vaccines, immunosuppressants, insulin, and botulinum neurotoxins [32, 33, 34, 35, 36]. Natural CPPs mainly come from organisms such as viruses, bacteria, fungi, and animals. These peptide segments have natural cell penetration ability. For example, the TAT protein of human immunodeficiency virus‐1 (HIV‐1) is a classic natural CPP, whose amino acid sequence contains multiple arginine residues that endow the TAT protein with strong cell penetration ability [37]. Pep‐1 can bind to negatively charged phospholipids on the cell membrane through electrostatic interactions, thereby promoting their transmembrane transport [38]. ANTP is derived from amino acids 43–58 of the Drosophila Antennapedia protein, and contains multiple arginine residues and hydrophobic amino acids such as phenylalanine and leucine. These hydrophobic amino acids help the peptide locate and insert into the cell membrane, thereby enhancing its penetration ability. ANTP can deliver various large molecular substances into cells and is widely used in gene therapy and drug delivery fields [39]. The R9 peptide is composed of nine arginine residues and has a very high positive charge density. It can enter cells through multiple pathways and exhibits strong cell penetration ability [40]. TP10 is a hybrid peptide composed of bee venom peptide and GALA peptide, and contains multiple lysine and arginine residues. It can bind to negatively charged phospholipids on the cell membrane through electrostatic interactions, thereby promoting its transmembrane transport [41]. KALA peptide is a designed amphiphilic peptide that can interact with the negative charge and the lipid bilayer on the cell membrane, allowing it to effectively penetrate the cell membrane [42]. The nuclear localization sequence (NLS) of primate polyoma virus (such as SV40) can utilize nuclear pore complexes to guide deoxyribonucleic acid (DNA) or proteins into the nucleus. Although these NLS do not fully conform to the characteristics of ideal CPPs, they still exhibit good membrane penetration ability when covalently linked with hydrophobic peptide sequences [43] (Table 3). The ease of CPP penetration into cell membranes suggests that it can be used to enhance the ability of PROTAC molecules to penetrate cell membranes [44]. For example, Naganuma et al. [45] developed a novel PROTAC molecule CPP/HDO–PROTAC coupled with heterologous double‐stranded oligonucleotides (HDO), significantly increasing its affinity for estrogen receptor alpha (ERα). Horibe et al. [46] utilized ANTP‐TPR to improve drug cell membrane penetration, promoting the degradation of tumor suppressor protein p53, protein kinase B (Akt), and cellular rapidly accelerated fibrosarcoma (cRaf), and enhancing the cytotoxic effect on breast cancer cells. These studies indicate that CPP is a feasible approach to address the issue of PROTAC cell membrane penetration, paving the way for its clinical application.

#### 3.2.2 Skin‐Penetrating Peptide

Skin‐penetrating peptide (SPP) is a type of peptide molecule that can penetrate the skin barrier and deliver drugs or other bioactive molecules directly to the deep layers of the skin or systemic circulatory system. They are typically rich in specific amino acids that determine their penetration ability and biological activity. Common transdermal peptides include transdermal Peptide 1(TD‐1), skin penetrating and cell entering (SPACE), and magainin. TD‐1 is a peptide composed of 11 amino acids, and is the first short peptide discovered through phage display technology to overcome the skin barrier, which can effectively carry protein drugs to penetrate the skin [47]. SPACE is a peptide composed of 11 amino acids. It can enhance the local transmission of large molecular substances such as hyaluronic acid, and can promote some proteins and small molecules to penetrate the stratum corneum [48]. Magainin is a peptide with antibacterial activity isolated from Xenopus laevis skin. It can penetrate the skin barrier and deliver antibacterial components directly to the deep layers of the skin, thereby exerting strong antibacterial effects [49]. SPPs have broad application prospects in the field of skin medication, which can significantly improve the transdermal absorption rate and bioavailability of drugs, providing more efficient and convenient treatment methods for patients with skin diseases (Table 3). For example, Zhu et al. [50] successfully increased the transdermal delivery capacity of drugs by 4.48 times by using TD‐1, achieving deeper penetration of drugs into the skin. This innovative method not only effectively inhibits the growth of melanoma in vivo, but also significantly induces apoptosis of tumor cells [50].

## 4 Mechanisms of Action of Peptide PROTACs

### 4.1 Target Protein Degradation via UPS

In the field of life sciences, the dynamic balance of proteins is crucial. Proteins are the main bearers of life activities and participate in various cellular functions, including metabolism, signal transduction, gene expression regulation, and so on. The dynamic balance of proteins, namely the balance between protein synthesis and degradation, is crucial for maintaining the normal function of cells [51]. When this balance is disrupted, it may lead to the occurrence of various diseases [52]. In the human body, new proteins are constantly produced, but the total protein level remains at a specific level, indicating the existence of a protein degradation system. The protein degradation system is the key to maintaining protein dynamic balance. In eukaryotic cells, protein degradation is mainly carried out through UPS [53]. This system consists of multiple components, mainly including Ub, Ub activating enzyme (E1), Ub conjugating enzyme (E2), Ub ligase (E3), and proteasome. Ub is a small‐molecule protein composed of 76 amino acid residues. It forms an isopeptide bond with the lysine residue of the target protein through its C‐terminal glycine residue, thereby connecting the Ub molecule to the target protein. E1 is responsible for activating Ub molecules to form high‐energy thioester bonds. E2 receives Ub molecules from E1 and transfers them to E3. E3 has high substrate specificity that can recognize specific target proteins and connect Ub molecules to target proteins [54]. Proteasome is a large multi subunit complex responsible for recognizing and degrading ubiquitinated proteins. The proteasome consists of 20S core particles and 19S regulatory particles. The 20S core particles have proteolytic activity, while the 19S regulatory particles are responsible for recognizing ubiquitinated proteins and unfolding them for degradation in the 20S core particles [55]. The UPS system is capable of identifying and degrading misfolded, damaged, aged, or unwanted proteins to maintain cellular homeostasis [56]. The characteristics of the UPS system in degrading proteins make it of great application value in the field of disease treatment, opening up new ideas for drug innovation. Just as human cells use the UPS system to finely regulate protein quantity, ensuring cell health and function, modern disease treatment technology is exploring and simulating this mechanism to achieve precise management of protein balance. Through in‐depth research on the protein degradation process of cells, researchers can develop new disease treatment ingredients that can mimic the functions of UPS systems, intelligently identifying and removing aging, damaged, unnecessary or harmful proteins in the body. Peptide PROTACs essentially act by binding target proteins to the UPS. The E3 ligase binding peptide of peptide PROTACs can bind to E3, causing the target protein to be spatially close to E3, resulting in ubiquitination modification of the target protein and enabling the target protein to enter the UPS, ultimately leading to its degradation through the UPS (Figure 2).

### 4.2 Advantages of Induced Proximity and Selectivity

The core mechanism of peptide PROTAC is to induce proximity, allowing specific target proteins to interact with enzymes (such as E3), thereby achieving selective degradation of target proteins. This technology has demonstrated unique advantages in inducing proximity and selectivity. Peptide PROTAC technology breaks through the traditional “occupancy‐driven” mode of small‐molecule inhibitors, which requires inhibitors to occupy the active site of the target protein in order to exert their effects. On the contrary, peptide PROTAC adopts an “event‐driven” mode, which constructs a bifunctional molecule that binds to the target protein on one end and recruits E3 ligase on the other end, thereby bringing the two together. This mechanism does not rely on the active site of the target protein, so it can target proteins with unexposed active sites or that are difficult to bind to small‐molecule inhibitors, greatly expanding the range of proteins that can be targeted. Once peptide PROTAC successfully induces the target protein to approach E3, the target protein will be labeled with a Ub tag, which will then be recognized and degraded by the proteasome [57].

Peptide PROTAC has multiple advantages in inducing selectivity. First, during the process of inducing target protein degradation, peptide PROTAC can specifically recognize and bind to the target protein and related E3. This sequence‐based recognition mechanism provides a basis for selectivity. Second, peptide PROTAC only needs to bind to the target protein to specifically “label” the target protein, greatly reducing the possibility of off‐target effects, thereby improving the selectivity for the target protein. Thirdly, peptide PROTAC can target proteins that are difficult for small‐molecule inhibitors to bind to. In the complex environment of cells, there are numerous proteins with different structures and functions. Peptide PROTAC can accurately find target proteins in complex environments without interfering with nontarget proteins, avoiding the impact on other normal physiological function‐related proteins [58]. In addition, there are many E3 in the human proteome. Peptide PROTAC can enhance the selectivity of target protein degradation by collaborating with specific E3 ligases [59].

### 4.3 Factors Influencing Efficacy and Selectivity

#### 4.3.1 Factors at the Molecular Design Level

The key to peptide PROTAC molecular design lies in selecting appropriate target protein binding peptides and E3 ligase binding peptides. The choice of these two peptide fragments directly determines the specificity and targeting of peptide PROTAC molecules. The affinity, selectivity, and binding stability of the two peptide fragments are key factors affecting the efficacy and selectivity of peptide PROTACs. In addition, the linker serves as a bridge between the target protein binding peptide and the E3 ligase binding peptide. Its length and flexibility are crucial for the formation of ternary complexes of peptide PROTAC molecules. Appropriate linker length and flexibility help PROTAC molecules form stable ternary complexes within cells, thereby improving efficacy and selectivity [60]. The physical and chemical properties of peptide PROTAC molecules, such as solubility, stability, permeability, and so on, also affect their efficacy and selectivity. For example, oral PROTAC drugs require good absorption properties, which are often influenced by factors such as molecular size, charge, and hydrophilicity. For instance, Zhang et al. [61] combined cancer‐targeted penetrating peptides with peptide PROTACs to enhance their cellular permeability. They developed 26 novel targeted penetrating peptides, among which C9C‐f (3Bta) and Cyclo‐C9C‐R exhibited excellent membrane permeability, targeting, and stability. After binding them with IMA–PROTAC, the intracellular IMA–PROTAC content significantly increased, which promoted Bcr–Abl protein degradation and tumor cell apoptosis [61]. Ma et al. [62] designed a novel peptide based PROTAC molecule DSARTC, which can stabilize the α‐helix and β‐sheet structures. Compared with its linear counterpart, DSARTC has higher stability and cell penetration ability. In animal models of prostate cancer, DSARTC effectively inhibits tumor growth and reduces AR and AR‐V7 levels, demonstrating its potential as a more effective and specific peptide PROTAC [62].

#### 4.3.2 Factors at the Level of the Biological Environment

The cell membrane is the first barrier for peptide PROTAC molecules to enter the cell. The size, shape, charge, and other characteristics of molecules can affect their cell membrane permeability. Peptide PROTAC molecules with poor cell membrane permeability are difficult to enter cells, thereby affecting their therapeutic efficacy [63]. The stability of peptide PROTAC molecules in vivo is the basis for their therapeutic effects. Unstable peptide PROTAC molecules may be degraded or inactivated in the body before reaching the target, thereby reducing therapeutic efficacy. Therefore, improving the in vivo stability of peptide PROTAC molecules is one of the key factors to enhance their therapeutic efficacy. For example, Hymel et al. [64] designed a peptide PROTAC that binds to β‐hairpins and found that β‐hairpins not only enhance the ability of peptide PROTACs to penetrate cell membranes, but also increase their stability, resulting in a longer lifespan of peptide PROTACs in cells. The metabolic pathway of peptide PROTAC molecules in vivo also affects their efficacy and selectivity. Different metabolic pathways may lead to differences in the half‐life, distribution, and excretion characteristics of peptide PROTAC molecules in vivo. Therefore, understanding the metabolic pathways of peptide PROTAC molecules is of great significance for optimizing their efficacy and selectivity. For certain peptide PROTAC molecules, repeated administration may trigger an immune response, leading to drug failure or adverse reactions. This immunogenicity risk needs to be fully evaluated in the design and clinical application of peptide PROTAC molecules. The tissue selectivity of peptide PROTAC molecules is another important factor affecting their efficacy and selectivity. The absorption, distribution, and excretion characteristics of peptide PROTAC molecules may vary among different tissues or organs. Therefore, peptide PROTAC molecules with tissue selectivity can more accurately target proteins in specific tissues or organs, thereby improving efficacy and reducing side effects. For example, Xu et al. [65] developed a peptide PROTAC drug targeting P21‐activated kinase 4 (PAK4), named PpD. This drug can efficiently and selectively degrade PAK4, effectively avoiding interference with other homologous proteins, demonstrating excellent targeting and specificity, and providing a new potential approach for the treatment of PAK4 related diseases [65].

## 5 Applications of Peptide PROTAC in Disease Treatment

### 5.1 Skin‐Related Conditions

In the field of skin health, maintaining the dynamic balance of proteins is the key to solving skin problems. As the largest organ in the human body, the skin is rich in various proteins both on its surface and in its deep layers. They not only construct the structural framework of the skin, but also participate in maintaining the skin barrier function, transmitting intercellular signals, and responding to external stimuli [66]. When the skin is exposed to external challenges such as ultraviolet radiation, environmental pollution, and increased stress, the dynamic balance of proteins is easily disrupted, leading to a series of problems such as aging, inflammation, and sensitivity in the skin [67]. Using drugs containing ingredients that accelerate the degradation of problematic proteins can help the skin recover and maintain its protein balance, thereby enhancing the skin's ability to self‐repair, reducing the occurrence of skin problems, and leaving the skin radiant and healthy [68]. In the field of precisely targeted skin disease treatment, peptide PROTAC technology has enormous potential for application. By precisely degrading specific proteins, peptide PROTAC technology is expected to fundamentally improve skin problems such as inflammation and pigmentation [69], driving innovation and upgrading in the skin disease treatment field and providing patients with more safe and effective skin disease treatment drugs [70].

#### 5.1.1 Pigmentation

The market for treating skin diseases such as pigmentation continues to grow globally, especially in Asia, where the market size and consumer demand are particularly significant. According to market research institutions, in recent years, global sales of pigmentation treatment drugs have steadily increased at an average annual rate of 7%, and this trend is expected to continue in the coming years. Among them, the Asian market occupies a dominant position, especially in China, Japan, and South Korea [71]. Patients have a strong demand for drugs treating pigmentation, which is closely related to the improvement of cultural aesthetics, skin color concepts, and sun protection awareness.

Peptide PROTAC can significantly treat pigmentation by precisely targeting specific proteins in the skin. The formation and distribution of melanin in the skin are key factors causing pigmentation. Peptide PROTAC can specifically bind to target proteins related to melanin synthesis, and polyubiquitinate the target proteins, allowing them to be recognized and degraded by proteasomes [72, 73]. Tyrosinase is a key protein in the synthesis of melanin, and its activity and expression level directly affect the depth of human skin color. Tyrosine is oxidized to dopa under the catalysis of tyrosinase, which is further oxidized to dopaquinone, and then undergoes a series of reactions to ultimately form melanin [74]. Chemical inhibitors are currently the most widely used tyrosinase inhibitors. This type of inhibitor inhibits melanin synthesis by binding to the active site of tyrosinase, blocking its catalytic activity. Common chemical inhibitors include vitamin C and its derivatives, arbutin, quercetin, and so on [75]. However, chemical inhibitors can cause significant harm to the human body. Physical methods mainly include phototherapy and laser therapy. These methods directly act on melanocytes or melanin particles through physical means, disrupting their structure or promoting their metabolic excretion, thereby achieving pigmentation treatment effects. However, these methods usually need to be carried out in professional medical institutions and come with certain risks and side effects. Biological inhibitors mainly include plant extracts and microbial fermentation drugs, which exert tyrosinase inhibitory effects through complex bioactive components. Compared with chemical inhibitors, biological inhibitors have the advantages of wide sources, high safety, and mild action. For example, green tea extract contains abundant tea polyphenols, such as catechins, which have significant antioxidant and tyrosinase inhibitory effects. They can eliminate free radicals, protect skin cells from oxidative damage, and inhibit the synthesis of melanin. The flavonoids in licorice extract have a wide range of biological activities, including anti‐inflammatory, antioxidant, and tyrosinase inhibition. They reduce melanin production by regulating the gene expression and enzyme activity of tyrosinase [76]. However, the whitening effect of existing biological inhibitors is limited, their efficacy is not specific, and the mechanism is unclear.

**FIGURE 3:**
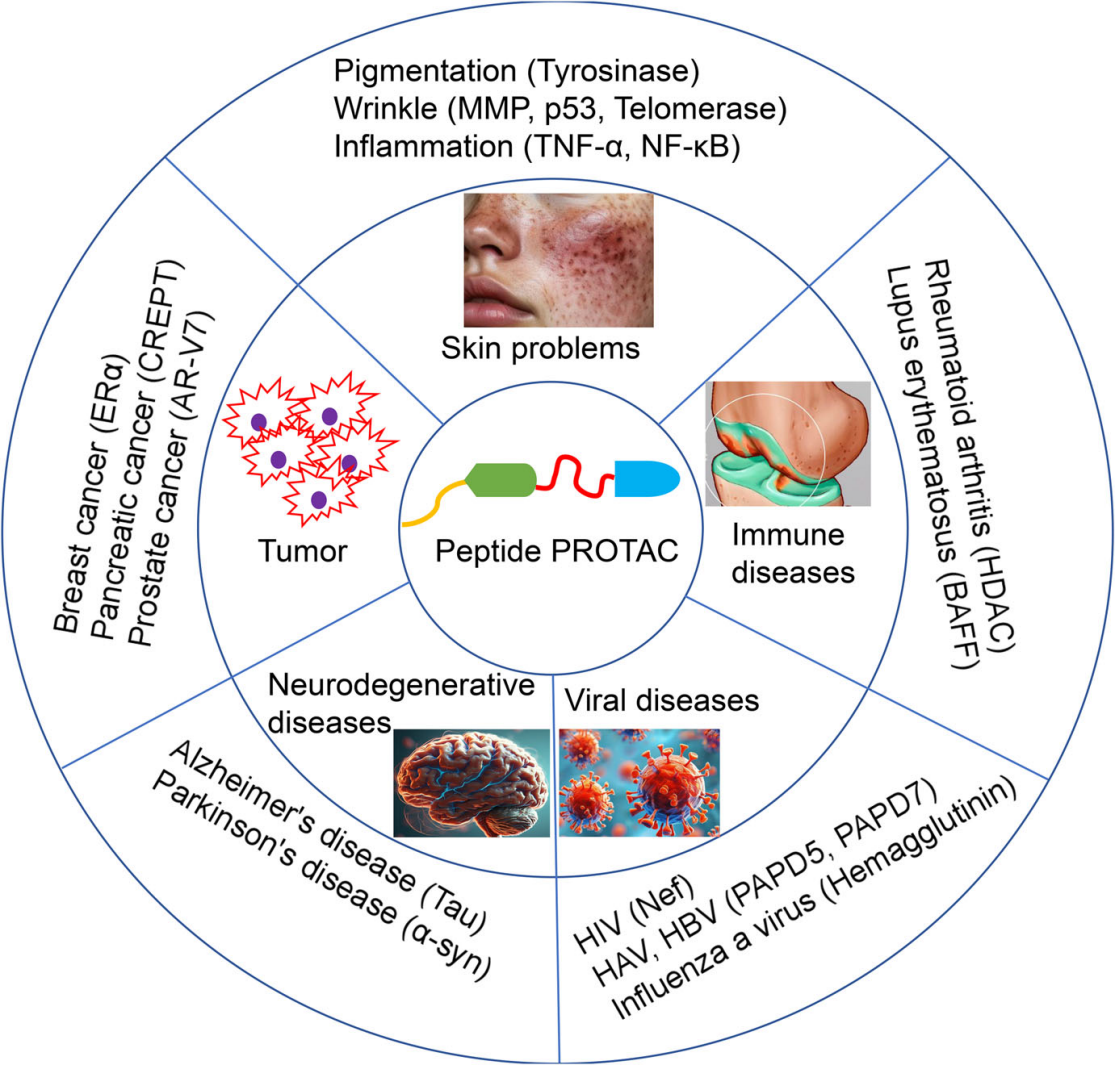
Examples of peptide PROTAC applications in different fields. The application areas of peptide PROTAC include skin problems, tumors, immune diseases, neurodegenerative diseases, and viral diseases [69, 132, 134].

The use of peptide PROTAC to degrade tyrosinase can reduce the generation of melanin, improve skin tone unevenness, and address pigmentation. The design focus of peptide PROTAC molecules with pigmentation treatment effects is to identify peptide sequences that can tightly bind to tyrosinase. This process typically involves a significant amount of screening and validation work. Scientists can use bioinformatics prediction, proteomic analysis, and high‐throughput screening techniques to identify potential peptide candidates. Subsequently, the affinity of these peptides for tyrosinase and their impact on tyrosinase activity are validated through in vitro experiments. Finally, peptide sequences with high affinity and effective inhibition of tyrosinase activity are selected as the ligand part of PROTAC molecules (Table 4 and Figure 3) [77]. In addition to the peptide target protein ligand, the linking peptide and E3 ligase binding peptide are also essential key elements in the design of peptide PROTAC molecules. The combination of the three forms a bifunctional peptide molecule that effectively binds to tyrosinase and E3 Ub ligase.

**TABLE 4:** Peptide sequences that can bind to pigmentation‐, aging‐, and inflammation‐related proteins.

Efficacy	Target protein	Target protein binding peptide	Mechanization
Pigmentation treatment	Tyrosinase	IQSPHFF [77]	With high affinity and effective inhibition of tyrosinase activity
		TASSDAWYR [77]	Hydrophobic interaction and hydrogen bonding of tyrosinase
		SAPFMPDAFFRNV [77]	Hydrophobic interaction and hydrogen bonding of tyrosinase
		YRSRKYSSWP [77]	Binding to tyrosinase
		MRSRERSSWP [77]	Binding to and inhibiting tyrosinase
		CNGVQPK [77]	Binding to and inhibiting tyrosinase
		LILVLLAI [77]	Binding to and inhibiting tyrosinase
		ECGYF [77]	Binding to and inhibiting tyrosinase
		TRCFRVCS [77]	Hydrophobic interaction and hydrogen bonding of tyrosinase
		AEDEPLLME [77]	Binding to and inhibiting tyrosinase
		YRSRKYSSWY [77]	Binding to and inhibiting tyrosinase
		YGGFMYSEKSQTPLVTLFKNAIIKNAHKKGE [77]	Binding to and inhibiting tyrosinase
Antiaging	MMP‐1	CTCVPPHPQTAFC [91]	Selecting the active domain of TIMP‐1; binding to MMP‐1
	MMP‐9	TFKEPVPDLC [92]	Binding to MMP‐9
	P53	LSQETFSDLWKLLPEN [95]	Bind to p53
	Aβ	Ac‐KQKLLFLEE‐NH2 [99]	Bind to p53
	Telomerase	AKWYDRRDYVF [102]	Selecting the conserved domain of p23; bind to p53
Anti‐inflammation	TNF‐α	EHMALTYPFRPP [109]	Binding to TNF‐α receptors
		ALWHWWH [109]	Binding to TNF‐α receptors
		TWLHWWA [109]	Binding to TNF‐α receptors
	NF‐κB	GRKKRRQRRRPPQCPVIRH [113]	Binding to the p50 subunit of NF‐κB; inhibiting the production of TNF‐α and IL‐6
		AAVALLPAVLLALLAPVQRKRQKLMP [114]	NF‐κB antagonist, binding to NF‐κB

Abbreviations: Aβ, β‐amyloid protein; IL‐6, interleukin‐6; MMPs, matrix metalloproteinases; NF‐κB, nuclear factor kappa B; p53, tumor suppressor protein; TNF‐α, tumor necrosis factor alpha.

The use of peptide PROTAC technology to degrade factors that affect keratinocyte formation and pigmentation, such as keratinocyte growth factor receptor (KGFR), can also improve skin pigment distribution and enhance whitening effects. KGFR, as an important type of cell membrane receptor, is widely expressed in the skin and various tissues, especially in the keratinocytes of the epidermis. It plays a crucial role in regulating processes such as cell proliferation, differentiation, migration, and apoptosis. However, when KGFR is excessively activated or expressed abnormally, it may lead to a series of skin problems, including but not limited to rough skin, keratin accumulation, and uneven pigmentation [78]]. These phenomena not only affect the appearance of the skin, but may also trigger skin diseases such as inflammation and acne. By degrading KGFR, peptide PROTAC technology can reduce abnormal proliferation and differentiation of keratinocytes from the source, thereby improving skin roughness, keratin accumulation, and other issues. Meanwhile, as KGFR also participates in regulating the synthesis and transport of melanin, its degradation has the potential to improve uneven pigmentation of the skin. Specifically, the degradation of KGFR can reduce the activation of melanocytes and decrease melanin synthesis; at the same time, it may also affect the transport and distribution of melanin, making skin pigmentation more uniform and natural. The target protein binding peptide of the PROTAC peptide targeting KGFR is currently known to include KGFR [79]. Finding peptides that can bind to KGFR is particularly important for developing whitening drugs. However, there are currently no reports of peptides that can bind to KGFR. This requires us to use computer‐aided design and other technologies to screen for new peptides.

The use of peptide PROTAC technology to degrade antioxidant enzyme inhibitors can enhance the inherent antioxidant capacity of the skin and reduce pigmentation caused by free radicals. Antioxidant enzymes are a type of enzyme that can clear free radicals in the body and prevent them from triggering oxidative stress reactions. In the skin, common antioxidant enzymes include superoxide dismutase (SOD), catalase (CAT), glutathione peroxidase (GPX), and so on. These enzymes protect skin cells from oxidative damage and maintain the health of the skin by catalyzing the breakdown of free radicals [80]. However, the activity of antioxidant enzymes is not always at its optimal state. Multiple factors, such as environmental factors (UV radiation, pollution), internal factors (aging, changes in hormone levels), and unhealthy lifestyle habits (smoking, alcohol abuse), can all lead to the inhibition of antioxidant enzyme activity, thereby weakening the skin's antioxidant capacity. When the antioxidant capacity is insufficient to resist the attack of free radicals, the skin will experience a series of problems such as pigmentation, wrinkles, sagging, and so on. Inhibitors of antioxidant enzymes refer to substances that can reduce the activity of antioxidant enzymes or hinder their normal function. These inhibitory factors may originate from the external environment or be produced by skin cells themselves. Common antioxidant enzyme inhibitors include metal ions (such as copper and iron), peroxides, inflammatory factors, and certain small‐molecule compounds. They inhibit the activity of antioxidant enzymes by binding to them, occupying enzyme active sites, or interfering with the interaction between enzymes and substrates, thereby exacerbating the oxidative stress response of the skin [81]. By degrading the inhibitory factors of antioxidant enzymes, peptide PROTAC technology can restore the activity of antioxidant enzymes and enhance their ability to scavenge free radicals. Specifically, when the peptide PROTAC molecule enters skin cells, its targeted ligand will specifically recognize and bind to antioxidant enzyme inhibitors. Subsequently, the inhibitory factor is guided to the UPS for degradation through the interaction between the linker and E3 Ub ligase recruiting molecules. This process not only reduces the number of inhibitory factors, but also releases inhibited antioxidant enzymes, allowing them to resume their antioxidant function. With the recovery and enhancement of antioxidant enzyme activity, skin cells can better resist the invasion of free radicals and reduce the occurrence of oxidative stress reactions. This not only helps reduce the formation of pigmentation, but also improves the overall health of the skin, making it smoother, more delicate, and elastic. The peptides that can bind to antioxidant enzyme inhibitors also require more screening work to be obtained.

Traditional whitening drugs often achieve whitening effects by inhibiting tyrosinase activity, blocking melanin synthesis pathways, or promoting melanin metabolism. However, these methods often take a long time to show significant results, and their effectiveness is often limited by the renewal cycle of skin cells and individual differences [82]. The peptide PROTAC technology is different, as it can directly degrade the source proteins that cause pigmentation, such as key enzymes or regulatory factors closely related to melanin synthesis. This direct and efficient mode of action significantly accelerates the whitening process, allowing patients to see noticeable skin color improvement in a shorter amount of time. After discontinuing traditional whitening drugs, due to the failure to fundamentally solve the problem of pigmentation, there is often a rebound phenomenon, meaning the skin color becomes darker again. The peptide PROTAC technology fundamentally weakens melanin production by degrading the source proteins that cause pigmentation. This solution strategy, which starts from the source, makes the whitening effect more long‐lasting and reduces the need for repeated use. Meanwhile, due to the high specificity of peptide PROTAC technology, it only degrades the target protein and does not interfere with the function of other normal cells, thus ensuring the safety and reliability of the whitening process [83]. Everyone's skin condition and whitening needs are different, and traditional whitening drugs often only provide one or a few fixed solutions. However, the emergence of peptide PROTAC technology has provided the possibility for the implementation of personalized pigmentation treatment solutions. By customizing designs based on factors such as genotype, skin color, and age for different populations, more precise and effective peptide PROTAC whitening drugs can be developed. These drugs can accurately target individual specific causes of pigmentation, achieving more significant and long‐lasting whitening effects. For example, it synthesized a series of PROTACs (D3‐D9) using Rhein as the target protein ligand, and the results showed that D6 can degrade MITF and inhibit the expression level of TYR in B16‐F10 cells, effectively inhibiting the production of melanin in zebrafish, and its whitening effect exceeds that of its precursor Rhein [84].

#### 5.1.2 Antiaging and Wrinkle Removal

Skin aging is a complex biological process involving various factors such as changes in gene expression, protein homeostasis imbalance, free radical damage, and reduction of collagen and elastin fibers. The formation of skin wrinkles is mainly attributed to structural changes in the dermis layer, especially the degradation of collagen and elastin fibers, as well as the decline in skin cell function (such as fibroblasts). Environmental factors such as ultraviolet radiation, smoking, chronic inflammation, and glycation reactions can accelerate this process [85]. The antiwrinkle strategy primarily combats skin aging by enhancing the skin's moisturizing ability, promoting collagen synthesis, inhibiting collagenase activity, and strengthening skin barrier function [86]. With the increasing trend of population aging, the issue of skin aging is receiving growing attention. Although traditional skin disease treatment methods such as antioxidants and moisturizers have certain effects, they often struggle to reverse the underlying mechanisms of skin aging. The emergence of peptide PROTAC technology provides a new approach to solving this problem. By targeting the degradation of proteins associated with skin aging and wrinkle formation, peptide PROTAC may achieve more direct and fundamental antiaging and antiwrinkle effects. For example, Jia et al. [87] developed PROTAC molecules targeting B‐cell lymphoma extra large (BCL‐X_L_) and BCL‐2, and confirmed their antiaging activity [87]. The biodegradability and low toxicity potential of peptide molecules make peptide PROTACs have broad application prospects in the field of skin disease treatment. Therefore, studying the mechanism of action and potential applications of peptide PROTAC in skin antiaging and wrinkle resistance has important scientific value and market potential for developing new skin disease treatment drugs and therapies.

One of the main causes of skin aging is a change in the structure of the dermis, which can be caused by many factors, such as the activation of matrix metalloproteinases (MMPs) by external factors, leading to excessive degradation of collagen and elastin in the dermis that support the skin structure, resulting in aging symptoms such as wrinkles and decreased skin elasticity. The most important MMPs are MMP‐1 and MMP‐9 (Figure 3) [88].

MMP‐1 is the main enzyme for degrading type I and type III collagen. When MMP‐1 is overexpressed, it specifically degrades extracellular matrix components, disrupts the normal structure of collagen fibers and elastic fibers, leading to aging symptoms such as wrinkles in the skin. Delaying skin aging by inhibiting MMP‐1 is an important means of antiaging. Inhibiting the activity of MMP‐1 enzyme can be achieved by inhibiting MMP‐1 enzyme itself, blocking MMP‐1 proenzyme activation, and blocking MMP‐1 synthesis at the gene level [89]. The most important endogenous MMP‐1 inhibitor in the human body is tissue inhibitor of metalloproteinases‐1 (TIMP‐1). TIMP‐1 can inhibit MMP‐1 activity by forming a stable molecular complex with MMP‐1, blocking the binding of MMP‐1 to the substrate, thereby inhibiting its activity, reducing the degradation of matrix collagen, and delaying skin aging [90]. Mihailovici et al. [91] confirmed that TIMP‐1 can inhibit MMP‐1 and promote collagen production [91]. By selecting the active domain of TIMP‐1, peptide segments that can tightly bind to MMP‐1 can be obtained (Table 4).

MMP9 is a zinc‐dependent endopeptidase that plays important roles in many biological processes, including tissue remodeling, embryonic development, wound healing, and angiogenesis. However, as age increases, the expression and activity of MMP9 may change, thereby being associated with the aging process and the occurrence and development of related diseases. MMP9 is a matrix MMP primarily responsible for degrading type IV collagen and gelatin on the basement membrane. It can also affect the aging process by regulating inflammatory responses, oxidative stress, and cell apoptosis [92]. Searching for peptide segments that can tightly bind to MMP‐9 can help us design antiaging peptide PROTACs (Table 4).

p53, also known as tumor suppressor protein, is an important transcription factor in cells that participates in regulating processes such as the cell cycle, DNA repair, and apoptosis. The p53 protein plays a crucial role in cell cycle regulation. When cells are stimulated by DNA damage, oxidative stress, malnutrition, or growth factor deficiency, p53 is activated. Activated p53 can bind to specific target gene promoters, promoting the expression of cell cycle checkpoint‐related genes such as p21. P21 can inhibit cyclin‐dependent kinase 2 (CDK2) and cyclin‐dependent kinase 4/6 (CDK4/6), thus preventing cyclin D and cyclin E from forming complexes with their CDK partners, thereby preventing cells from entering the S phase for DNA replication from the G1 phase. P53 can also induce cell apoptosis or cell cycle arrest to prevent DNA‐damaged cells from continuing to proliferate and to prevent the occurrence of tumors [93]. During skin aging, the activity and function of p53 undergo changes. Under normal circumstances, p53 monitors DNA damage to prevent abnormal cell proliferation caused by gene mutations. As age increases, DNA damage accumulates and the activation frequency of p53 increases, leading to an increase in cell cycle arrest and cellular aging. The cell aging mediated by p53 can prevent the proliferation of abnormal cells, thereby preventing the occurrence of tumors, but at the same time, it can also lead to a decrease in the renewal ability of skin cells, affecting skin repair and regeneration. Excessive activation of p53 may lead to excessive apoptosis of skin cells, exacerbating epidermal thinning and collagen loss during skin aging [94]. Therefore, the precise regulation of p53 is crucial for maintaining skin health and preventing aging. The application of peptide PROTAC technology provides new possibilities for precise intervention in skin aging. Peptide PROTACs have the potential to become a novel therapy for treating p53‐related skin aging. By using peptide PROTAC‐specific degradation technology to regulate p53 levels, peptide PROTACs may help restore the normal physiological state of the skin, restore normal cell proliferation and function, improve the microstructure and function of the skin, reduce wrinkles, enhance skin elasticity and radiance, and improve overall skin health. Finding peptides that can tightly bind to p53 is the key to designing p53‐targeted antiaging peptide PROTACs. Allen et al. [95] developed a series of peptides and found that p53AD1 (LSQETFSDLWKLLPEN) has a strong ability to bind to p53 (Table 4) [95].

β‐Amyloid protein (Aβ) plays an important role in skin aging, and its abnormal metabolism and accumulation are one of the important causes of skin aging. Understanding the biological function of Aβ and its mechanism of action in the skin is of great significance for developing new antiaging strategies for the skin. In the skin, Aβ mainly exists in nerve endings, sweat glands, and hair follicles, participating in nerve signal transmission and maintaining skin barrier function. The metabolic process of Aβ involves multiple enzymes, such as α‐, β‐, and γ‐secretases, which participate in the cleavage of Aβ precursor proteins and generate Aβ fragments of different lengths. Under normal circumstances, the activity of these enzymes and the clearance mechanism of Aβ maintain a dynamic balance, preventing the accumulation of Aβ [96]. Recent studies have found that Aβ plays an important role in skin aging. In the skin, the accumulation of Aβ can lead to an imbalance in cellular homeostasis, affecting the synthesis of collagen and elastin fibers, thereby accelerating skin sagging and wrinkle formation. Aβ may also promote the process of skin aging by increasing oxidative stress and inflammatory response. As people age, the physiological functions of the skin gradually decline, including decreased elasticity and moisturizing ability, as well as weakened repair ability. The accumulation of Aβ is considered a key factor in this process. The deposition of Aβ can interfere with the normal function of skin cells, such as affecting the differentiation and proliferation of keratinocytes, as well as the collagen synthesis of fibroblasts [97]. Aβ can also induce oxidative stress responses in the skin, produce a large amount of free radicals, damage cell membranes and DNA, and lead to cellular dysfunction. Aβ can also activate inflammatory reactions, release inflammatory factors, promote skin inflammation and fibrosis, and accelerate the process of skin aging [98]. The abnormal metabolism and accumulation of Aβ are one of the important causes of skin aging. Understanding the biological function of Aβ and its mechanism of action in the skin is of great significance for developing new antiaging strategies for the skin. Peptide PROTAC technology will show significant potential in regulating Aβ levels. The design of peptide PROTAC molecules specifically targeting Aβ can promote the degradation of Aβ in skin cells, thereby promoting collagen synthesis and improving skin barrier function. Takahashi and Mihara [99] developed a peptide that could bind to Aβ, with the sequence “Ac‐KQKLLFLEE‐NH 2,” which provided assistance in designing PROTAC molecules targeting Aβ (Table 4) [99].

Telomerase is a crucial reverse transcriptase, whose main function is to maintain the telomeric DNA at the ends of chromosomes from damage. With each cell division, telomeres gradually shorten until they reach a critical length, leading the cell into an aging state. The activity of telomerase is closely related to the proliferation ability of skin cells. During the process of skin aging, telomerase activity decreases, the ability of skin cells to divide weakens, leading to loss of elasticity, wrinkles, and other signs of aging [100]. During the process of skin aging, changes in telomerase activity not only affect cell proliferation, but may also affect gene expression and signaling pathways, such as the regulation of aging‐related genes such as p53 and pRb [101]. Peptide PROTAC can regulate the level of telomerase by specifically binding and degrading it, thereby slowing down the process of skin aging. Finding peptides that can tightly bind to telomerase can help us design peptide PROTAC molecules targeting telomerase. However, peptides that can tightly bind to telomerase have hardly been reported. However, p23 is a protein that can bind to telomerase [102]. If the peptide segment of the p23 conserved domain is extracted (for example, AKWYDRRDYVF) for the synthesis of peptide PROTAC molecules (Table 4), it may achieve the purpose of ubiquitination and degradation of telomerase.

#### 5.1.3 Anti‐inflammation

Skin inflammation is a common pathological state in skin diseases, including eczema, dermatitis, acne, and other types. These diseases are often accompanied by symptoms such as redness, itching, and flaking, which seriously affect the quality of life of patients. Currently, anti‐inflammatory treatment for the skin mainly relies on steroid drugs, nonsteroidal anti‐inflammatory drugs, and immune modulators, but long‐term use of these drugs may result in side effects such as skin atrophy, pigmentation, and dependence. Therefore, finding safer, more effective, and less side‐effect treatment methods has become a hot topic in the field of skin anti‐inflammatory research [103].

Peptide PROTAC exhibits excellent anti‐inflammatory effects through its unique targeted degradation mechanism. It can accurately identify and degrade target proteins related to inflammatory responses, such as inflammatory mediators and signal transduction proteins, thereby blocking the transmission and amplification of inflammatory signals. By degrading these inflammation‐related proteins, peptide PROTAC can significantly alleviate inflammatory symptoms, such as redness, swelling, itching, and pain in the skin. At the same time, it can also promote the repair and regeneration of skin cells, accelerating the healing of inflammatory wounds. This mechanism makes peptide PROTAC an ideal anti‐inflammatory ingredient for treating skin diseases, effectively addressing various skin inflammation issues. For example, Eccleston successfully applied PROTAC technology in autoimmune skin diseases. Clinical studies have found that PROTAC technology can alleviate excessive autoimmunity, thereby reducing the symptoms of autoimmune skin diseases [104]. Peptide PROTAC has the potential ability to target multiple inflammatory factors, such as nuclear factor kappa B (NF‐κB) and tumor necrosis factor alpha (TNF‐α). These factors play a central role in the inflammatory response of the skin, leading to the exacerbation of skin inflammation by regulating the expression of inflammatory genes. The intervention of peptide PROTAC may effectively reduce the levels of these proteins, thereby inhibiting the inflammatory signaling pathway and reducing the inflammatory response of the skin.

TNF‐α is a cytokine produced by immune cells such as monocytes, macrophages, and certain types of T cells when stimulated by pathogens. TNF‐α plays a crucial role in immune regulation, inflammatory responses, and cell death. It binds to receptors on the cell surface, triggering signaling pathways that affect gene expression, promote inflammatory responses, inhibit tumor growth, and regulate immune responses. Under physiological conditions, TNF‐α helps maintain the balance of the immune system, but excessive or sustained activation of TNF‐α may lead to chronic inflammation and autoimmune diseases [105]. In dermatology, TNF‐α is a key molecule in the skin immune response and is involved in the pathogenesis of various skin diseases, such as psoriasis and contact dermatitis. Overexpression and persistent presence of TNF‐α may lead to excessive inflammatory responses, causing skin redness, itching, and flaking. It also participates in the formation of acne by inducing excessive activity of sebaceous glands and abnormal proliferation of hair follicle keratinocytes. The binding of TNF‐α receptor‐associated factor 4 (TRAF4) to deubiquitinase USP10 can induce p53 instability and promote fibroblast proliferation. Meanwhile, TNF‐α induced polymorphism of TNFAIP3 may be associated with scleroderma [106]. Regulating the activity and expression levels of TNF‐α is a key strategy for treating and preventing skin diseases [107]. Reducing excessive TNF‐α activity through inhibitors or antibodies can effectively alleviate skin inflammation. For example, TNF‐α antagonists have shown significant efficacy in the treatment of psoriasis [108]. Targeting TNF‐α therapy has become an important treatment direction in dermatology, especially for patients who have poor response or intolerance to traditional treatment methods. Searching for the peptides that can tightly bind to TNF‐α receptors is a prerequisite for designing anti‐inflammatory peptide PROTAC (Figure 3). Researchers have used phage screening technology to identify peptides that can bind to TNF‐α receptors, which provides assistance in developing peptide PROTAC molecules targeting TNF‐α (Table 4) [109].

NF‐κB is a key transcription factor involved in cellular responses to external stimuli, including inflammation, immune responses, and cell proliferation. The normal function of the NF‐κB pathway is crucial in the skin, as it regulates the expression of multiple genes through a series of complex signal transduction processes. Usually, NF‐κB binds to I κB protein in the cytoplasm outside the nucleus and remains inactive. When cells are stimulated by pathogens, ultraviolet radiation, or chemicals, I κB protein is degraded, releasing NF‐κB, allowing it to enter the nucleus and activate target gene transcription [110]. NF‐κB plays multiple roles in skin cells. It affects the differentiation of keratinocytes and maintains skin barrier function. In fibroblasts, NF‐κB participates in the synthesis of collagen and elastin fibers, and has a direct impact on the structure and elasticity of the skin. It also regulates the activity of skin immune cells such as Langerhans cells and macrophages, participating in the skin's immune defense [111]]. NF‐κB plays a central role in the inflammatory response of the skin. It can initiate the expression of a series of proinflammatory genes, including cytokines, chemokines, and adhesion molecules, leading to the recruitment of immune cells and exacerbation of inflammatory responses. In skin diseases such as psoriasis, eczema, and acne, excessive activation of NF‐κB leads to chronic inflammation and skin lesions [112]. Therefore, NF‐κB has become a potential target for the treatment of skin inflammatory diseases. By designing peptide PROTAC molecules targeting NF‐κB receptors or their signaling pathways, regulation of NF‐κB levels can be achieved, which may inhibit skin inflammation and delay skin aging (Figure 3). For example, Wang et al. [113] designed a peptide that can bind to the p50 subunit of NF‐κB, with the sequence: GRKKRRQRRRPPQCPVIRH. It could effectively inhibit the production of NF‐κB and interleukin‐6 (IL‐6) in the human leukemic cell line (THP‐1) and could treat PMA‐induced ear edema and  zymosan A‐induced peritonitis in mice [113]. SN50 is an antagonist of NF‐κB, formed by connecting the hydrophobic region of the Kaposi fibroblast growth factor signal peptide with the NLS of NF‐κB p50, and exerts its effect by inhibiting the translocation of NF‐κB. It can penetrate into cells, significantly inhibit the activation of NF‐κB in the body, and alleviate inflammatory reactions and related diseases (Table 4) [114].

### 5.2 Oncology

Many tumor‐associated proteins lack effective drug‐binding pockets, making traditional small‐molecule inhibitors difficult to act effectively. The effective intervention, high selectivity, and low toxicity of peptide PROTACs on these “untreatable” targets enable them to overcome the limitations of traditional treatment methods and bring new hope to cancer patients. There are some proteins in cells that are closely related to the occurrence of cancer, and their abnormal expression or dysfunction is often a key factor in tumor development. Scientists can efficiently reduce the levels of these proteins in cancer cells by specifically identifying and degrading them, thereby achieving the goal of inhibiting tumor growth and spread and providing new strategies and means for cancer treatment.

#### 5.2.1 Degradation of Tumor Target Proteins by Peptide PROTAC

The precise targeting and degradation of key proteins related to tumor development using peptide PROTAC technology can effectively inhibit tumor growth and development. Estrogen receptor α (ERα) is an effective target for the treatment of ER+ breast cancer (Figure 3). Jiang et al. [115] designed a peptide PROTAC targeting ERα by linking a stable peptide ERα modulator (TD‐PERM) cross‐linked with N‐terminal aspartic acid to a pentapeptide binding to VHL E3 Ub ligase complex. The resulting heterodimeric peptide (TD‐PROTAC) could selectively degrade ERα [115]. Akt is also an important cancer target. Researchers have prepared p‐PROTAC (CPP tria‐PR), which can degrade Akt protein in vitro [116]. Forkhead box M1 (FOXM1) is a proliferation‐related transcription factor overexpressed in various human tumors and is considered a key driver of tumor occurrence and progression, as well as a potential target for anticancer therapy. However, currently, peptide PROTACs targeting FOXM1 have not been extensively studied. Wang et al. [117] constructed a novel FOXM1–PROTAC targeting FOXM1, which could effectively enter cells and degrade FOXM1 protein, significantly inhibiting the survival, migration, and invasion abilities of various cancer cell lines. At the same time, it inhibited tumor growth in human epithelial cell line 2 (HepG2) and MDA‐MB‐231 cell xenograft mouse models and was nontoxic to normal tissues. In addition, FOXM1–PROTAC reduced glucose metabolism in cancer cells by downregulating glucose transporter 1 (GLUT1) and programmed cell death‐1 (PD‐L1) expression, indicating that FOXM1 is involved in cancer metabolism and immune regulation. Therefore, the biological targeted degradation of FOXM1 is a safe and promising strategy for treating cancers with FOXM1 overexpression [117]. Cell cycle‐related and expression‐elevated protein in tumor (CREPT) is highly expressed in cancer and can promote the proliferation of pancreatic cancer cells (Figure 3). The permeable p‐PROTAC (PRTC) prepared by Ma et al. [118] could effectively degrade CREPT protein, inhibit the proliferation of pancreatic cancer cells, and significantly suppress tumor growth in the mouse xenograft model without obvious side effects. This method provided new targets and strategies for cancer treatment [118]. Jin et al. [4] reported a new peptide PROTAC (xStAx VHLL) that could effectively inhibit mouse Wnt‐dependent colon cancer by degrading β‐catenin. This discovery confirmed that constrained conformation could enhance the drug properties of peptide PROTACs, providing new targets and strategies for cancer treatment [4]. AR splicing variant 7 (AR‐V7) is a key drug target for prostate cancer (CRPC) (Figure 3), but currently there are no effective drugs targeting it. Ma et al. [119] developed a novel peptide PROTAC drug based on AR DBD homodimeric structure, which could induce AR‐V7 degradation [119]. PD‐L1 is an important target for cervical cancer. Shi developed a PROTAC based on cyclic peptides, which could induce the degradation of palmitoyltransferase in human cervical cancer cells and effectively reduce the level of PD‐L1 [15]. Liu et al. [120] developed a novel peptide‐based RNA binding protein‐PROTAC (RALY–PROTAC), which could serve as a degrader of RALY protein and demonstrate potential for treating RALY‐overexpressing liver cancer, providing a new mechanism and drug option for liver cancer treatment (Table 5) [120].

**TABLE 5:** Potential tumor targets of peptide PROTACs.

Category	Targets	Mechanisms
Breast cancer	ERα 115]	TD‐PERM binds to VHL, targeting ER α
	GRP94, CDK1/4 [121]	Degrading GRP94 and CDK1/4 in tumor cells, leading to cell cycle arrest and induces cell apoptosis
Pancreatic cancer	CREPT [118]	Degrading CREPT protein, inhibiting pancreatic cancer cell proliferation
Intestinal cancer	β‐Catenin [4]	Degrading β‐catenin, inhibiting Wnt‐dependent colorectal cancer
Prostate cancer	AR‐V7 [119]	Inducing AR‐V7 degradation
Cervical cancer	PD‐L1 [15]	Inducing degradation of palmitoyltransferase in human cervical cancer cells and effectively reducing the expression of PD‐L1
Liver cancer	RALY 120]	Degradation products of RALY protein, showing potential for treating RALY overexpressing liver cancer
Leukemia	BCL‐X_L_ [123]	Effectively inhibiting tumor growth without causing thrombocytopenia
	STAT3 [125]	Inducing rapid degradation of STAT3, exhibiting nanomolar cell growth inhibitory activity against leukemia and lymphoma cell lines
	STAT5 [126]	Inducing STAT5 depletion
Lymphoma	BCL6 [127]	Degradation of BCL6 in diffuse large B‐cell lymphoma (DLBCL) cell lines
Other tumors	Akt [116]	Preparing p‐PROTAC (CPP tria‐PR), to degrade Akt
	FOXM1 [117]	Degrading FOXM1, inhibiting the survival, migration, and invasion abilities of various cancer cells
	p53‐R175H [122]	Degradation of carcinogenic mutation p53‐R175H
	SHP2 [124]	Significantly inhibiting Hela cell growth, induce SHP2 degradation and cell apoptosis
	KRAS [131]	Inducing degradation of KRAS
	NAMPT [132]	Degradation of NAMPT, inhibition of MDSC, and improvement of antitumor efficacy

Abbrevations: Akt, protein kinase B; AR‐V7, androgen receptor splicing variant 7; BCL‐XL, B‐cell lymphoma; BCL6, B‐cell lymphoma 6; CDK1/4, cyclin‐dependent kinase 1/4; CREPT, cell cycle‐related and expression‐elevated protein in tumour; ERα, estrogen receptor; FOXM1, Forkhead box M1; GRP94, glucose‐regulated protein 4; KRAS, Kirsten rat sarcoma viral oncogene homolog; NAMPT, Nicotinamide phosphoribosyltransferase; p53‐R175H, mutant p53 in saos‐2; PD‐L1, programmed cell death‐1; RALY, RNA‐binding protein; SHP2, Serine hydroxyl phosphatase 2; STAT3, Signal transducer and activator of transcription factor 3; STAT5, Signal transducer and activator of transcription factor 5; TD‐PERM, peptide ERα modulator.

#### 5.2.2 Other Potential Tumor Targets for Peptide PROTACs

The mechanism of action of peptide PROTAC is similar to that of small‐molecule PROTAC molecules. In theory, the targets of small‐molecule PROTACs are also potential targets for peptide PROTACs. For example, Gan et al. [121] designed and synthesized a PROTAC molecule based on triptolide, which could selectively degrade glucose‐regulated protein 4 (GRP94) and CDK1/4 targets in tumor cells, leading to cell cycle arrest and apoptosis in breast cancer cells [121]. Kong et al. [122] developed a PROTAC degrader called dp53m, which could specifically degrade the oncogenic mutant 53 in saos‐2 (p53‐R175H) while preserving the function of wild‐type p53. This degrader has shown significant antitumor effects on p53‐R175H‐driven cancer cells in both in vitro and in vivo experiments without toxicity. In addition, dp53m could significantly enhance the sensitivity of these cancer cells to the chemotherapy drug cisplatin. These findings provided strong evidence for the potential value of dp53m in p53‐R175H‐driven cancer therapy [122]. B‐cell lymphoma (BCL)‐X_L_ is a key target for cancer treatment, but the traditional drug ABT263 is limited due to thrombocytopenia. Khan et al. [123] developed a PROTAC (DT2216) that could target BCL‐X_L_. DT2216 is more effective than ABT263 in treating BCL‐X_L_‐dependent leukemia and cancer cells, and has less platelet toxicity. In vivo experiments showed that DT2216 could effectively inhibit tumor growth without causing thrombocytopenia [123]. Serine hydroxyl phosphatase 2 (SHP2), as a member of the protein tyrosine phosphatases (PTPs) family associated with various cancers, is an important target for drug development. Zheng et al. [124] developed a PROTAC molecule SP4 targeting SHP2 using cereblon (CRBN), which was formed by linking pomalidomide with SHP099, a conformational inhibitor of SHP2. SP4 exhibited a 100‐fold increase in activity compared with SHP099, significantly inhibiting HeLa cell growth, inducing SHP2 degradation and cell apoptosis [124]. Signal transducer and activator of transcription factor 3 (STAT3) is also an important target for cancer treatment but is hard to target. Zhou et al. [125] discovered a small‐molecule SD‐36 based on the PROTAC concept. SD‐36 could induce rapid degradation of STAT3 at low nanomolar concentrations without degrading other STAT proteins and has nanomolar cell growth inhibitory activity against leukemia and lymphoma cell lines with high levels of phosphorylated STAT3. Low‐dose SD‐36 could lead to complete degradation of STAT3 protein in tumor tissue and achieved complete and persistent tumor regression at well‐tolerated doses in tumor models, indicating that SD‐36 is a potent, selective, and effective STAT3‐degrading agent [125]. STAT5 is an attractive target for cancer therapy. Kaneshige et al. [126] developed a PROTAC‐based degradation agent, AK‐2292, which could effectively induce STAT5A/B protein degradation. AK‐2292 effectively induced STAT5 depletion in normal mouse tissues and human chronic myeloid leukemia (CML) xenograft tissues, and achieved tumor regression at well‐tolerated doses in CML xenograft mouse models [126]. B‐cell lymphoma 6 (BCL6) inhibition is a promising mechanism for treating hematological cancers. McCoull et al. [127] developed a PROTAC molecule that significantly degraded BCL6 in many diffuse large B‐cell lymphoma (DLBCL) cell lines [127]. Kim et al. [128] designed, synthesized, and evaluated a series of PROTAC molecules composed of bicalutamide analogs and thalidomide as novel AR degraders. Among them, compound 13b could successfully target and degrade AR in AR‐positive cancer cells, which might become a useful chemical probe for studying AR‐dependent cancer cells and a potential therapeutic drug candidate for prostate cancer [128]. Xiang et al. [129] discovered an oral bioavailable PROTAC AR degrader, ARD‐2585, which exhibited extremely low DC50 values and could effectively inhibit cell growth in cell lines with AR gene amplification and mutation. ARD‐2585 also exhibited excellent pharmacokinetics and oral bioavailability in mice and was more effective than existing drugs in inhibiting tumor growth [129]. Hu et al. [130] designed an ER degradation agent, ERD‐308, based on the PROTAC concept. ERD‐308 showed a low DC50 value in an ER+ breast cancer cell line and could induce a high proportion of ER degradation. Compared with approved ER‐degrading agents, ERD‐308 showed a more complete degradation ability of ER and was more effective in inhibiting cell proliferation [130]. Kirsten rat sarcoma viral oncogene homolog (KRAS) mutation is a common target in human cancers. Bond et al. [131] developed a PROTAC molecule, LC‐2, which showed rapid and sustained degradation of KRAS, demonstrating that PROTAC‐mediated degradation was a feasible option for reducing KRAS levels, providing a new strategy for cancer treatment [131]. Nicotinamide phosphoribosyltransferase (NAMPT) is a promising target for cancer treatment. Wu et al. [132] demonstrated that NAMPT could promote myeloid‐derived suppressor cell (MDSC) amplification and suppress antitumor immunity. PROTAC A7 was identified as an effective degrader of NAMPT based on PROTAC technology, capable of degrading intracellular NAMPT and reducing extracellular NAMPT secretion. In vivo experiments showed that PROTAC A7 could effectively degrade NAMPT, inhibit MDSCs, and improv antitumor efficacy. Its anticancer activity was superior to that of NAMPT enzyme inhibitors, providing a new direction for NAMPT‐targeted therapy (Table 5) [132].

### 5.3 Other Potential Therapeutic Areas

#### 5.3.1 Immune‐Related Diseases

Immune disorders are a type of disease caused by abnormal activation or dysfunction of the immune system, including rheumatoid arthritis and systemic lupus erythematosus. The characteristic of these diseases is that the immune system mistakenly attacks its own tissues and organs, leading to chronic inflammation and tissue damage [133]. Peptide PROTAC technology can precisely regulate key molecules of the immune system, thereby achieving more effective treatment of immune‐related diseases. For example, targeted degradation of histone deacetylase (HDAC) can alleviate synovial cell inflammation, cell invasion, and bone erosion, and improve rheumatoid arthritis (Figure 3) [134]. Interleukin‐1 (IL‐1) receptor associated kinase 3 (IRAK3) is a pseudokinase member of the IRAK family, which does not rely on kinase activity but instead utilizes the noncatalytic mechanism of its protein structure to transmit signals. Research has shown that IRAK3 may play an important role in the pathogenesis of osteoarthritis (OA), providing a new potential target for the use of peptide PROTACs in the treatment of OA [135]. In 2020, Degorce et al. [136] first developed a protein degrading agent for IRAK3. They quickly screened a series of high‐quality IRAK3 ligands by utilizing by‐products from IRAK4 precursor synthesis. After evaluating the binding affinity with IRAK3, cis‐pyrrolotriazine was ultimately selected as the ligand to develop PROTAC by binding with CRBN. This was the first reported PROTAC targeting IRAK3, laying the foundation for the development of peptide PROTACs targeting IRAK3 [136]. In the treatment of systemic lupus erythematosus, peptide PROTAC can target the degradation of B cell activating factor receptors on the surface of B cells, reducing B cell activation and the production of autoantibodies, thereby alleviating the condition (Figure 3) [137]. Overexpression of prostaglandin D2 (PGD2) in cells is closely related to various diseases, including allergic diseases and Duchenne muscular dystrophy. Hematopoietic prostaglandin D synthase (H‐PGDS) is a key enzyme in the synthesis of PGD2 and is therefore considered a major therapeutic target for these diseases [138, 139]. Yokoo et al. [140] released the first PROTAC drug in 2021 that utilized the UPS to degrade H‐PGDS. They connected TFC‐007 (a ligand for H‐PGDS) to pomalidomide (a ligand for CRBN) via a PEG linker to synthesize the first generation of H‐PGDS PROTAC drugs and found that its degradation efficiency of H‐PGDS could reach over 80% at 10 nM (DC50 = 17.3 pM), effectively inhibiting the production of H‐PGDS in KU812 cells [140]. Yokoo et al. [141] further optimized the PROTAC molecule and found that after removing the PEG linker, the PROTAC molecule could still form ternary complexes with higher selectivity and degradation efficiency. In vivo experiments also confirmed that the optimized PROTAC molecule had a better degradation effect on H‐PGDS. In addition, biological experiments have shown that PROTAC drugs targeting H‐PGDS can also inhibit the production of cytokines such as IL‐6, suggesting that they may have good therapeutic effects on inflammatory diseases [141]. The use of peptide PROTACs to inhibit H‐PGDS is expected to treat allergic inflammation. IRAK4 is a key regulatory protein of immune signaling, which can be expressed in various cells and is responsible for transmitting signals from Toll‐like receptors (TLRs) and IL‐1 family receptors (mainly including IL‐R1, IL‐18R, and IL‐33 receptor ST2) [142]. When the ligand activates the TLR or IL‐1 receptor, downstream MyD88 is recruited to the receptor and forms a multimeric protein complex with IRAK family proteins. The assembly of this complex promotes the autophosphorylation and activation of IRAK4, which in turn activates downstream MAPK and NF‐κB pathways, ultimately leading to inflammation. IRAK4 plays an important role in diseases such as asthma and chronic obstructive pulmonary disease [143]. Dai et al. synthesized a novel PROTAC by combining highly selective IRAK4 inhibitors with thalidomide. After treating human embryonic kidney (HEK293T) cells with a concentration of 405 nM for 24 h, this PROTAC could reduce the intracellular IRAK4 content by more than 90%. In addition, they further validated the function of the PROTAC by stimulating THP‐1 cell models with lipopolysaccharide (LPS), confirming that the PROTAC targeting IRAK4 could weaken NF‐κB activation and reduce IL‐6 production [144]. This provides ideas for the development of peptide PROTACs targeting IRAK4. Receptor‐interacting serine/threonine kinase 2 (RIPK2) is a downstream signaling molecule of nucleotide‐binding oligomerization domain 1 (NOD1), nucleotide‐binding oligomerization domain 2 (NOD2), and TLRs, which is mainly expressed in antigen‐presenting cells such as dendritic cells and macrophages, and can promote the release of proinflammatory factors such as TNF‐α, IL‐6, IL‐12, and so on. Recent experiments and clinical studies have shown that the activation of RIPK2 is closely related to the development of autoimmune diseases, especially inflammatory bowel disease (IBD) [145]. The expression of RIPK2 and related signaling molecules is enhanced in the colonic mucosa of IBD patients, and blocking RIPK2 can slow down the development of colitis. These findings reveal that RIPK2 activation is the immunological basis for the onset of IBD, suggesting that it may be a therapeutic target for IBD [146]. Bondeson et al. [147] explored the effects of different linkers on the structure–activity relationship to determine the optimal linker, and then applied it to RIPK2–PROTAC. Experiments showed that the PROTAC could completely degrade RIPK2 after 4 h without toxicity, making it an excellent intracellular RIPK2 degrading agent [147]. This points the way for the development of peptide PROTAC targeting RIPK2. Cluster of differentiation 147 (CD147) is a transmembrane glycoprotein belonging to the immunoglobulin superfamily, widely expressed in white blood cells, platelets, epithelial cells, and endothelial cells. It is not only an inducer of extracellular matrix MMPs, but also interacts with cyclophilins [148], angiotensin converting enzyme 2 [149], and integrin [150], and participates in various physiological and pathological activities. CD147 plays a crucial role in inflammation and immune response, closely related to the pathogenesis of various inflammation‐related diseases such as asthma‐mediated lung inflammation, rheumatoid arthritis, and multiple sclerosis [151]. Zhou et al. [152] screened a highly efficient PROTAC for degrading CD147 by finely adjusting the structure of the natural product pseudo lauric acid B (PAB) and linker, with a degradation efficiency of over 90% at a concentration of 40 µM. Cell and animal experiments confirmed that the therapeutic effect of PROTAC was significantly better than that of PAB [152].

#### 5.3.2 Neurodegenerative Diseases

Neurodegenerative diseases are a type of disease characterized by the gradual loss of function and death of neurons, leading to a gradual decline in cognitive, motor, and other physiological functions, which include Alzheimer's disease (AD), Parkinson's disease (PD), Huntington's disease, and amyotrophic lateral sclerosis [153, 154]. At present, the treatment of neurodegenerative diseases primarily focuses on relieving symptoms and delaying disease progression. In neurodegenerative diseases, the application of peptide PROTAC technology mainly focuses on clearing pathological proteins and regulating neuroprotective signaling pathways. AD is a neurodegenerative disease of the central nervous system. Episodic memory impairment is its initial symptom, which may be accompanied by cognitive, behavioral, and executive impairments later on [155]. Ultimately, it will lead to severe dementia in patients, which has a very serious impact on their daily lives [156]. Abnormal aggregation of Aβ and tau proteins is the main pathological feature of AD (Figure 3) [157, 158]. Peptide PROTAC can reduce neuronal damage by targeting and degrading these pathological proteins [159]. Tau protein is a neuronal microtubule‐binding protein, produced by alternative splicing of the microtubule‐associated protein tau (MAPT) gene [160], playing a role in regulating microtubule stability and axonal transport [161]. It can be misfolded and form aggregates in neurodegenerative diseases such as AD [162], and can spread aggregates between cells [163]. Its high phosphorylation is a hallmark of AD and related diseases [164]. Based on this, preventing tau protein aggregation has become an important strategy for treating tau protein‐mediated neurodegenerative diseases. Chu et al. [165] designed and synthesized the first peptide‐based PROTAC molecule TH006 targeting tau protein. They determined that TH006 had a high binding affinity with tau protein through fluorescence polarization analysis. In vitro experiments showed that TH006 could successfully penetrate cells and induce endogenous tau protein degradation, reducing the neurotoxicity of Aβ, with a dose‐ and time‐dependent effect [165]. Lu et al. [166] developed a peptide PROTAC molecule for degradation of tau proteins. Experiments showed that the peptide PROTAC molecule had strong binding affinity with Kelch‐like ECH‐associated protein‐1 and tau protein, and could significantly promote tau protein degradation in human neuroblastoma SH‐SY5Y cells. This discovery provided a new pathway for tau protein degradation and was expected to become a promising strategy for the treatment of neurodegenerative diseases [166]. Wang et al. [167] developed a novel PROTAC molecule (C004019) that could simultaneously recruit tau and the E3 Ub ligase Vhl. C004019 exhibited strong tau clearance effects in both cellular and mouse models and significantly improved synaptic and cognitive functions [167]. Glycogen synthase kinase 3 (GSK‐3) is a multifunctional serine/threonine protein kinase belonging to the phosphotransferase family and is considered a therapeutic target for various diseases. It has a high content in the brain, especially in neurons and astrocytes [168], and its expression level increases with age [169]. Research has shown that GSK‐3 can promote tau protein phosphorylation and Aβ peptide production [170], which has a significant impact on the development of AD [171]. Jiang et al. [172] designed and synthesized a series of small‐molecule PROTACs based on CRBN for the degradation of GSK‐3β. Among them, mycoplasma hominis PG21 exhibited good GSK‐3β degradation ability and a moderate half‐maximal inhibitory concentration (IC50) value, and protected HT‐22 hippocampal neuronal cells from glutamate‐induced cell death. This was the first PROTAC instance that could degrade GSK‐3β, providing potential candidate drugs for the study of GSK‐3β‐related diseases and laying the groundwork for the development of peptide PROTAC molecules targeting GSK‐3β [172]. PD is a neurodegenerative disorder that primarily affects the motor system. Its pathological features are the gradual degeneration and loss of dopaminergic neurons in the substantia nigra of the midbrain, accompanied by the formation of inclusions such as α‐synuclein (α‐syn), parkin, and Ub [173]. Abnormal aggregation of α‐syn is the main pathological feature in PD (Figure 3). Peptide PROTAC can reduce neuronal death by targeting α‐syn to induce its degradation [174]. Wen et al. [175] designed and synthesized a PROTAC molecule that could specifically bind to α‐syn. Among them, compound 5 had the most significant degradation effect. It could also inhibit the increase in ROS levels and protect cells from α‐syn toxicity. This provided an experimental basis for the treatment of neurodegenerative diseases related to α‐syn [175].

#### 5.3.3 Viral Diseases

Viral diseases are one of the major challenges facing global public health. Although significant progress has been made in the development of vaccines and antiviral drugs in recent years, issues such as high virus mutation rates, host immune evasion, and drug resistance still constrain the effectiveness of treatment [176]. In antiviral therapy, peptide PROTAC can target key proteins in the viral replication cycle, such as viral polymerases, proteases, and so on. HIV is a virus that can attack the human immune system. HIV mainly invades the human immune system, especially CD4+T lymphocytes, which play a crucial role in the human immune system. HIV patients may experience various infections and tumors, and in severe cases, it can lead to death. There is currently no effective drug to cure HIV [177]. HIV‐1 integrase is one of the key enzymes involved in HIV replication. Peptide PROTAC can recruit E3 Ub ligase to degrade HIV‐1 integrase, thereby inhibiting HIV replication [178]. The Nef cofactor of HIV‐1 is important for the virus lifecycle, as it can promote immune escape and virus persistence, and is a potential target for antiretroviral drugs (Figure 3). Emert‐Sedlak et al. [179] developed a PROTAC for targeted degradation of Nef by coupling a Nef‐binding compound with a Ub E3 ligase ligand. This PROTAC can ubiquitinate and degrade Nef, effectively inhibiting HIV replication [179]. The viral infectivity factor (Vif) is also widely recognized as a therapeutic target for HIV‐1 infected patients. Luo et al. [180] designed a series of PROTACs, among which L15 could significantly degrade Vif and exhibit antiviral activity. Molecular dynamics simulations indicated that L15, Vif, and E3 ligases formed stable ternary complexes [180]. Hepatitis viruses are the pathogens that cause viral hepatitis, mainly including hepatitis A virus (HAV) and hepatitis B virus (HBV) [181]. RG7834 can inhibit HAV and HBV by suppressing dihydroquinolinone of polyadenylation polymerases 5 and 7 (PAPD5 and PAPD7) (Figure 3). Li et al. [182] reported a PROTAC molecule based on RG7834, which could degrade PAPD5, thereby inhibiting HAV and HBV [182]. Li et al. [183] designed and synthesized some PROTACs based on pentacyclic triterpenoids. Among them, V3 could effectively degrade hemagglutinin (Figure 3), with a median degradation concentration of 1.44 µM. V3 has broad‐spectrum antiviral activity against influenza A virus. Further analysis showed that V3 was cross‐linked with the new targets Asn15, Thr31, and Asn27 of hemagglutinin, providing a new direction for the development of anti‐influenza drugs [183]. Si et al. [184] introduced a removable proteasome‐targeting domain into the viral genome to create a PROTAC virus, which could be attenuated by the host degradation mechanism after infection and trigger a strong and widespread immune response, providing a new approach for the preparation of attenuated live vaccines [184]. These studies provide new ideas for the treatment of viral diseases using peptide PROTAC.

### 5.4 The Drug Development Process of Peptide PROTAC

**FIGURE 4:**
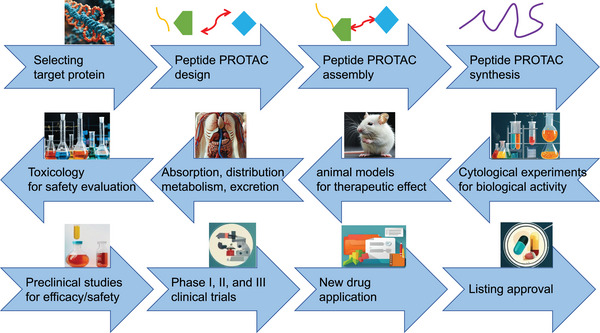
Flowchart of the drug development process of peptide PROTAC. The drug development process of peptide PROTAC mainly includes selecting target protein, peptide PROTAC design, peptide PROTAC assembly, peptide PROTAC synthesis, cytological experiments for biological activity, animal models for therapeutic effect, absorption, distribution, metabolism, and excretion of peptide PROTAC, toxicology for safety evaluation, preclinical studies for efficacy/safety, phase I, II, and III clinical trials, new drug application, and listing approval [188, 191].

The development of peptide PROTAC drugs is a complex and multistage process: (1) Determining the target of disease treatment, that is, to select the target protein. The target protein is a protein closely related to the occurrence and development of diseases [185]. (2) Designing the molecular structure of peptide PROTAC, including the selection of peptide segments for delivery enhancement peptides, target protein binding peptides, E3 ligase binding peptides, and linker peptides [186]. (3) Combining peptide segments into a whole. (4) Synthesizing complete peptide PROTAC molecules through chemical or biosynthetic methods [187]. (5) Validating the biological activity of peptide PROTAC molecules through cytological experiments, namely their degradation effect on target proteins [188]. (6) Further validating the biological activity of peptide PROTAC molecules in vivo using animal models and evaluating their therapeutic effect on diseases [188]. (7) Studying the absorption, distribution, metabolism, and excretion characteristics of peptide PROTAC molecules in vivo [189]. (8) Evaluating the potential toxicity of peptide PROTAC molecules to animal organs and systems through toxicological studies, ensuring the safety of the drug [189]. (9) Evaluating the efficacy and safety of peptide PROTAC molecules through preclinical studies (Table 6) [190]. (10) Application for clinical trials: Submitting a clinical trial application to regulatory agencies and conducting Phase I, II, and III clinical trials to evaluate the safety, efficacy, and optimal dosage of peptide PROTAC molecules after obtaining approval (Table 6) [191]. (11) New drug application: Submitting new drug applications to regulatory agencies based on clinical trial results [192]. (12) Market approval: After approval by regulatory authorities, peptide PROTAC drugs will be allowed to be sold and used in the market (Figure 4). Post‐market drugs also require phase IV clinical trials to monitor their long‐term safety, efficacy, and potential side effects, providing a basis for improving the drug [193].

**TABLE 6:** Relevant preclinical animal experiments and clinical trials.

Phase	Segment	Purpose
Preclinical animal experiments	Pharmacodynamic research	Evaluating the mode of action and efficacy of drugs on living organisms [188] Determining the main pharmacological parameters of the drug, such as the onset time, duration of action, and intensity of action [188]
	Pharmacokinetics study	Investigating the absorption, distribution, metabolism, and excretion processes of drugs in living organisms [188] Understanding the dynamic changes of drugs in the body provides a basis for developing reasonable dosing plans and predicting drug behavior in the human body [188]
	Toxicological research	Evaluating drug safety, including acute toxicity, long‐term toxicity, carcinogenicity, mutagenicity, and reproductive toxicity [190]
Clinical trials	Phase I clinical trial	Evaluating the safety and tolerability of drugs [191] Observing the side effects and adverse reactions of the medication [191]
	Phase II clinical trial	Evaluating the effectiveness and safety of drugs in specific patient populations [191]
	Phase III clinical trial	The final hurdle before drug launch Ensuring the safety and effectiveness of the drug when widely used [191]
	Phase IV clinical trial	Studies conducted after a drug is launched Monitoring the safety and efficacy of the drug during long‐term use Identifying potential side effects and adverse reactions Providing a basis for the improvement and rational use of drugs [193]

## 6 Challenges in the Application of Peptide PROTAC Molecules

When exploring the potential applications of peptide PROTAC molecules in the field of disease treatment, we have to face a series of challenges in practical applications. These challenges not only involve the depth and breadth of scientific research, but also relate to the commercialization process of drugs and the user experience of patients. This review will further analyze the challenges of peptide PROTAC molecular applications and look forward to possible solutions and future development trends.

### 6.1 Complexity of Synthesis and Purification

**FIGURE 5:**
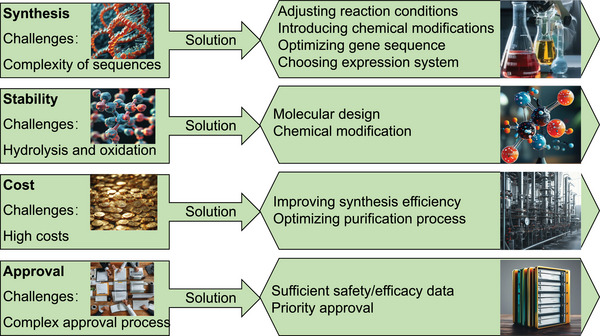
Overview of the main challenges and potential solutions for current peptide PROTAC technologies. The main challenges faced by peptide PROTAC technology include synthesis, stability, cost, and approval [201].

The chemical synthesis of peptide PROTAC molecules is a complex and intricate process that requires highly skilled chemical synthesis techniques and precise molecular design. Due to the fact that peptides themselves are composed of multiple amino acids, the diversity and complexity of their sequences make the synthesis process highly prone to errors. In addition, PROTAC molecules also need to precisely connect peptide ligands with linkers, E3 recruiters, and other components to form complete molecules with specific structures and functions. Each step in this process requires strict condition control and precise operation, and even a slight mistake may lead to synthesis failure or a decrease in product purity [194]. The synthesis of peptide PROTAC can be accelerated by changing the reaction system, such as using mixed solvents, to promote the condensation reaction. Adjusting the reaction conditions, such as the reaction temperature, pH value, and the type and concentration of salt ions, can also improve the efficiency of peptide synthesis. In addition, introducing chemically modified peptide chains or using fragment synthesis methods can promote peptide synthesis as well [195]. If peptide PROTACs are synthesized through genetic engineering technology, it is necessary to optimize the gene sequences encoding the target peptides and increase their expression levels. This includes adjusting elements such as promoters, enhancers, and terminators, as well as selecting efficient expression systems. The growth and metabolic environment of cells also need to be improved, such as providing sufficient nutrients, optimizing the culture medium formula, and controlling the culture conditions. In addition, choosing efficient expression systems such as yeast, bacteria, or mammalian cells is also very helpful in improving the synthesis efficiency of peptide PROTACs. Furthermore, introducing inducers or changing environmental conditions can help to precisely control the synthesis time and expression level of peptide PROTACs, thereby improving their synthesis efficiency (Figure 5) [196].

Even if the peptide PROTAC molecule is successfully synthesized, its purification is still a challenging task. Due to the potential production of various by‐products and impurities during the synthesis process, these impurities often have chemical properties similar to the target molecule and are difficult to remove through simple separation methods. Therefore, efficient purification technologies such as high‐performance liquid chromatography and gel filtration chromatography are needed to ensure the purity and stability of the target molecules [197]. Optimizing chromatographic techniques, including selecting appropriate chromatographic packing materials, adjusting elution conditions and flow rates, can improve the efficiency of peptide PROTAC separation and purification. In addition, new technologies such as electrophoresis, membrane filtration, and affinity purification can be attempted to improve the efficiency of peptide PROTAC separation and purification. Furthermore, developing new separation and purification methods, such as peptide easy clean (PEC) purification technology based on chemical selective separation, may address the limitations of traditional methods (Figure 5) [198].

### 6.2 Stability and Bioavailability Issues

Enzymatic hydrolysis and oxidation in living organisms are one of the main reasons for the decreased stability and bioavailability of peptide PROTAC molecules. Enzymatic hydrolysis can break down the peptide bond structure, degrading them into small molecular fragments; oxidation may alter the chemical properties of peptides, causing them to lose their original biological activity. Therefore, how to improve the enzymatic hydrolysis resistance and antioxidant capacity of peptide PROTAC molecules has become an urgent problem to be solved [63]. By rational molecular design and chemical modification, the antihydrolysis and antioxidant abilities of peptide molecules can be effectively enhanced. For example, by replacing specific amino acids or altering the sequence of peptide chains, the likelihood of peptide molecules being recognized by enzymes can be reduced, thereby improving their resistance to enzymatic hydrolysis. Introducing specific chemical groups, such as antioxidant groups, can enhance the antioxidant properties of peptide molecules (Figure 5) [199].

### 6.3 Production Costs and Pricing Considerations

The synthesis and purification process of peptide PROTAC molecules is complex and costly, which directly leads to an increase in production costs and product prices. The high price may affect the competitiveness of peptide PROTAC molecules in the market. Therefore, how to reduce production costs and improve production efficiency has become an important issue in the commercialization process of peptide PROTAC molecules. Improving the efficiency of synthesizing peptide PROTAC can greatly reduce the cost of peptide PROTAC, especially for methods utilizing synthetic biology technology to develop peptide PROTAC. For example, Shi et al. [200] successfully expressed apidaecin‐type antimicrobial peptides in Pichia pastoris and increased their expression level to 1.76 g/L, which greatly reduced the production cost [200]. Moreover, optimizing the purification process to improve the yield of peptide PROTAC can also reduce costs (Figure 5).

### 6.4 Regulatory and Approval Challenges

When applying peptide PROTAC molecules to drugs, strict regulatory policies and approval processes are also required. There are differences in regulatory standards and requirements for drugs in different countries and regions, which makes the commercialization process of peptide PROTAC molecules more complex and challenging. In addition, as the peptide PROTAC molecule belongs to a novel bioactive molecule, its safety and efficacy evaluation requires more rigorous and comprehensive data support. Therefore, applying for relevant approvals may require more time and resources [201]. To accelerate the market launch process of peptide PROTAC drugs, sufficient safety and efficacy evaluation data should be provided. Some countries prioritize the approval of innovative therapeutic drugs, thereby promoting their rapid market launch (Figure 5).

## 7 Future Perspectives in Peptide PROTAC Research and Development

### 7.1 Innovations in Peptide Design and Delivery Systems

**FIGURE 6:**
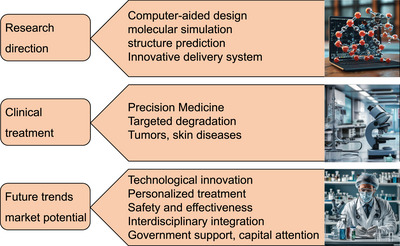
Forecast map of future research directions and market prospects. Research directions mainly include computer‐aided design, molecular simulation, structure prediction, and innovative delivery systems. Clinical treatment primarily focuses on precision medicine for tumors, skin diseases, and so on. Future trends mainly involve technological innovation, personalized treatment, enhancing safety and effectiveness, and interdisciplinary integration. With government and capital support, the market prospects for peptide PROTACs are promising [207].

The design flexibility of peptide PROTAC gives it a unique advantage in targeting multiple issues. By adjusting the sequence and structure of cell membrane penetrating peptides, target protein binding peptides, linker peptides, and E3 ligase binding peptide fragments, peptide PROTAC can be customized for different types of problems. This high degree of customization makes peptide PROTAC have broad application prospects in the field of precisely targeted disease treatment. Computer‐aided design technology has become an innovative direction for peptide PROTAC molecule design. For example, Wang et al. [202] utilized structural biology and computer protein structure prediction techniques to design novel peptide PROTAC molecules. These peptide PROTACs had higher targeting ability than controls [202]. Chen et al. [203] innovatively designed SP PROTACs based on Staphylococcal peptide, which combined the cellular uptake ability and hydrolysis stability of Staphylococcal peptide with the target degradation effect of PROTACs. SP–PROTAC could effectively kill cancer cells and inhibit tumor progression by promoting degradation of murine double minute 2 (MDM2) and murine double minute X (MDMX) and persistent activation of p53 [203]. Thapa et al. [204] developed CRBN‐dependent PROTACs, which could destroy harmful proteins through the UPS, and had promising prospects for the treatment of hematological malignancies and solid tumors. The development of CRBN‐based PROTACs is expected to provide new strategies and biomarkers for cancer treatment [204]. Innovative delivery systems can significantly improve the delivery efficiency of peptide PROTAC molecules. Constructing a delivery system using peptide coupling technology is one of the innovative directions. Peptides have good biocompatibility and modifiability, and can be coupled with PROTAC molecules through chemical bonds. This coupling method can improve the solubility, stability, and other properties of PROTAC, which is helpful for its delivery in vivo. For example, He et al. [205] innovatively designed the iRGD–PROTAC conjugation strategy, using the tumor‐penetrating cyclic peptide iRGD to deliver PROTAC to the depth of breast cancer tissue. The iRGD–PROTAC conjugate iPR has excellent water solubility, tumor targeting, and tissue permeability, significantly improving the anti‐breast cancer effect [205]. In addition, biodegradable nano delivery systems can improve the delivery efficiency of peptide PROTACs. These nanocarriers can protect drug stability, improve bioavailability, circulation time, and tissue‐specific targeting, thereby enhancing therapeutic efficacy. Combining nanotechnology with peptide PROTACs can solve the problems of targeted delivery and specific accumulation. For example, Li et al. [206] loaded anticancer peptides into lipid nanoparticles to achieve selective damage to cancer cells and avoid off‐target effects (Figure 6) [206].

### 7.2 Clinical Translation and Therapeutic Potential

Peptide PROTAC has shown great potential in clinical treatment as an innovative therapy. Compared with traditional disease treatment techniques, PROTAC disease treatment represents truly precise disease treatment. Especially, peptide PROTAC has set a precedent for its application in precise disease treatment. Traditional disease treatment techniques may not be able to accurately address the root cause of specific problems, and the results may not always be satisfactory. The mechanism of traditional disease treatment drugs is unclear, and the effect is not significant. The emergence of PROTAC disease treatment, especially peptide PROTAC disease treatment technology, has completely changed this situation. The occurrence of diseases often depends on specific signaling pathways and proteins. By degrading these key proteins, peptide PROTAC can effectively achieve the goal of treating diseases. This makes peptide PROTAC have great application prospects in the treatment of tumors, skin diseases, autoimmune diseases, and so on. The rise of peptide PROTAC technology not only marks a major innovation in the biomedical field, but also brings new hope to many patients with refractory diseases. We believe that in the near future, peptide PROTAC will become an important means of treating these diseases, providing more precise and effective treatment services for patients (Figure 6).

### 7.3 Emerging Trends and Market Outlook

Looking ahead to the future, the development direction of peptide PROTAC will pay more attention to technological innovation, personalized treatment, safety and efficacy improvement, and interdisciplinary cooperation. With the development of research, peptide PROTAC technology will continue to innovate and upgrade. On the one hand, researchers will strive to optimize the design of peptide PROTAC molecules, improve their degradation efficiency, stability, specificity, and bioavailability, achieve customized design, and expand the application scope of peptide PROTAC. Peptide PROTAC technology provides strong support for personalized therapy and precision medicine. Due to the significant differences in disease type, severity, and individual differences among different patients, traditional “one size fits all” treatment methods often struggle to achieve optimal results. Peptide PROTAC can precisely degrade specific disease‐related proteins, thereby achieving personalized treatment for diseases. In the future, researchers will use high‐throughput technologies such as genomics, proteomics, and metabolomics to conduct more comprehensive molecular typing of patients, in order to determine the most suitable peptide PROTAC treatment strategy. This will help improve treatment effectiveness, reduce side effects, and promote further development of precision medicine. Although peptide PROTAC technology has shown good safety and tolerability in preliminary clinical trials, the safety and efficacy of long‐term use still need further validation. In the future, researchers will strengthen toxicological studies on peptide PROTAC drugs, evaluate their potential side effects and long‐term effects. At the same time, artificial intelligence and machine learning technologies will be utilized to predict and optimize the efficacy of peptide PROTAC drugs, in order to further enhance their safety and effectiveness. Peptide PROTAC technology can be combined with other synthetic biology techniques to enhance its value. For example, HIV can integrate its genetic material into the human genome for replication, making it very difficult to cure. If peptide PROTAC technology is combined with CRISPR/Cas9 gene editing technology, the pathogenic gene can be knocked out by CRISPR/Cas9 gene editing technology while the disease‐related proteins can be degraded by peptide PROTAC technology, potentially achieving fundamental treatment of AIDS [207]. The development of peptide PROTAC technology also relies on interdisciplinary cooperation and innovation. In the future, researchers will strengthen cooperation with experts in multiple fields such as chemistry, physics, biology, pharmacy, computing, materials science, and engineering to jointly address the challenges faced by peptide PROTAC technology (Figure 6).

With the intensification of global population aging and the continuous increase in chronic diseases, the market demand for new, efficient, and safe therapeutic drugs is growing day by day. Peptide PROTAC technology is gradually becoming a hot topic in the field of drug development due to its unique therapeutic mechanism and wide application prospects. Especially in the fields of tumors, skin diseases, and autoimmune diseases, peptide PROTAC technology is expected to bring new treatment options to patients. Meanwhile, the innovation and breakthrough of peptide PROTAC technology will lay a solid foundation for its future market prospects. Peptide PROTAC technology will also attract strong support from the government and attention from the capital market, providing sufficient funding support for its research and application (Figure 6) In addition, the development of computer science and artificial intelligence technology will continuously expand the application scope of peptide PROTAC technology and inject new vitality into its future market prospects. Market competition will also promote the industrialization and commercialization of peptide PROTAC technology, bringing benefits and convenience to more patients. With the continuous advancement of technology and the expansion of the market, peptide PROTAC technology will usher in a new era of true precision medicine for humanity.

## Author Contributions

Youmin Zhu was responsible for conceptualization, data curation, formal analysis, investigation, methodology, project administration, validation, writing the original draft, review, and editing. Yu Dai was responsible for conceptualization, investigation, methodology, project administration, validation, writing the original draft, review, and editing. Yuncai Tian was responsible for project administration and supervision. All authors approved this manuscript for publication.

## Conflicts of Interest

Authors Youmin Zhu, Yu Dai, and Yuncai Tian are employees in Shanghai Zhizhenzhichen Technologies Co., Ltd. Shanghai Zhizhenzhichen Technologies Co., Ltd. has no role in preparation of the manuscript or decision to publish.

## Ethics Statement

The authors have nothing to report.

## Data Availability

The data included in this study are available upon request from the corresponding author.
